# WT1^+^ glomerular parietal epithelial progenitors promote renal proximal tubule regeneration after severe acute kidney injury

**DOI:** 10.7150/thno.79326

**Published:** 2023-02-21

**Authors:** Xizhen Hong, Hao Nie, Juan Deng, Shiting Liang, Liting Chen, Jing Li, Siqiao Gong, Guobao Wang, Wei Zuo, Fanfan Hou, Fujian Zhang

**Affiliations:** 1 Division of Nephrology, Nanfang Hospital, Southern Medical University, Guangzhou, China; 2 State Key Laboratory of Organ Failure Research, Southern Medical University, Guangzhou, China; 3 National Clinical Research Center of Kidney Diseases, Nanfang Hospital, Guangzhou, China; 4 Guangdong Provincial Clinical Research Center for Kidney Disease, Guangzhou, China; 5 Guangdong Provincial Key Laboratory of Renal Failure Research, Guangzhou, China; 6 Guangzhou Regenerative Medicine and Health Guangdong Laboratory, 510005 Guangzhou, China; 7 East Hospital, School of Medicine, Tongji University, Shanghai 200120, China; 8 Kiangnan Stem Cell Institute, Zhejiang 311300, China; 9 Division of Nephrology, Second Affiliated Hospital of Zhejiang University School of Medicine, No.88, Jiefang Road, Shangcheng District, Hangzhou, Zhejiang, 310009, China

**Keywords:** WT1, parietal epithelial cells, renal progenitors, regeneration, acute kidney injury

## Abstract

**Rationale:** Mammalian renal proximal tubules can partially regenerate after acute kidney injury (AKI). However, cells participating in the renal proximal tubule regeneration remain to be elucidated. Wilms' tumor 1 (WT1) expresses in a subtype of glomeruli parietal epithelial cells (PECs) in adult kidneys, it remains unclear whether these WT1^+^ PECs play a role in renal regeneration/repair after AKI.

**Methods:** Ischemia-reperfusion injury (IRI) mouse model was used to investigate the expression pattern of WT1 in the kidney after severe AKI. Conditional deletion of WT1 gene mice were generated using Pax8^CreERT2^ and WT1^fl/fl^ mice to examine the function of WT1. Then, genetic cell lineage tracing and single-cell RNA sequencing were performed to illustrate that WT1^+^ PECs develop into WT1^+^ proximal tubular epithelial cells (PTECs). Furthermore, *in vitro* clonogenicity, direct differentiation analysis and *in vivo* transplantation were used to reveal the stem cell-like properties of these WT1^+^ PECs.

**Results:** The expression of WT1 protein in PECs and PTECs was increased after severe AKI. Conditional deletion of WT1 gene in PTECs and PECs aggravated renal tubular injury after severe AKI. WT1^+^ PECs develop into WT1^+^ PTECs via the transient scattered tubular cell stage, and these WT1^+^ PECs possess specific stem cell-like properties.

**Conclusions:** We discovered a group of WT1^+^ PECs that promote renal proximal tubule regeneration/repair after severe AKI, and the expression of WT1 in PECs and PTECs is essential for renal proximal tubule regeneration after severe kidney injury.

## Introduction

Acute kidney injury, characterized by abrupt decrease in urine output and a rapid increase in serum creatinine 1, is a heterogeneous clinical syndrome with multiple etiologies, variable pathogenesis, and diverse outcomes 2. The tubular epithelium is known to have regenerative potential depending on the degree of injury 3. An emerging body of literature supports a role for endogenous surviving epithelial cells to replenish proximal tubular epithelial cells (PTECs) after injury 4-9. Meanwhile, numerous studies have demonstrated that renal progenitor cells exist in the adult kidney and participate in the repair process of tubule injury 10-15. However, it is still debatable whether specialized progenitors are involved in the regeneration/repair of injured renal proximal tubules.

Parietal epithelial cells (PECs) are a monolayer of heterogeneous cell populations lining Bowman's capsule and continuous with neighboring podocytes and PTECs 16. Besides the classical flat PECs, the neglected cuboidal PEC and intermediate PEC subgroups were identified recently 17. Because of their diversity, the role of PECs in health and disease conditions is not clearly understood. Studies using cell lineage tracing techniques suggested that PECs may serve as adult podocyte progenitors 18-22. Several lines of evidence indicated that PECs induced crescent formation in the development of glomerulosclerosis and proliferative lesions 23-25. In glomerular diseases, the intermediate PECs near the glomerular tuft became more numerous, proliferated faster than classical flat PECs, and formed early lesions preferentially 17. Researchers using *in vitro* clonal analysis and xenograft experiments also claimed that PECs are committed toward tubular lineage and have regenerative potential in the renal proximal tubule repair process after injury 11, 14. Nevertheless, there is no direct evidence to demonstrate that PECs are involved in regenerating renal proximal tubules *in vivo*.

Cells expressing CD24 and CD133, scattered throughout the human renal proximal tubules, were reported to increase resistance to AKI, and enable rapid repopulation of the tubules 13. These scattered tubular cells (STCs) phenotypically contain less cytoplasm, fewer mitochondria, and no brush borders 26, and co-express vimentin, kim1, annexin A3, CD44, and Akap12 in rodents and humans 5, 13, 26. The STC phenotype was considered a transient regenerative response pattern of renal proximal tubule repair after injury 5, 26, 27. The protein expression patterns of STCs and PECs were shown to have striking similarities 26, 28. However, the relationship between STCs and PECs and whether they represent a fixed progenitor population in the kidney needs further investigation.

The mammalian Wilms' tumor 1 (WT1) gene encodes a zinc finger transcription factor required for normal kidney development 29 and is highly expressed in mature podocytes. It has been demonstrated that WT1 plays a vital role in mature podocyte survival and regeneration 30-32. Results of the emerging single-cell RNA sequencing (scRNA-seq) have revealed that WT1 was not entirely podocyte specific, but also expressed in a subtype of PECs in adult kidneys. Nevertheless, the functional relevance and underlying mechanisms of WT1^+^ PECs in renal physiology and pathophysiology remain elusive.

In this study, we used WT1 conditional knockout mice to investigate the protective role of WT1 in AKI progression. We illustrated that WT1^+^ PECs developed to PTECs using genetic cell lineage tracing and scRNA-seq. Furthermore, we showed the stem cell-like properties of WT1^+^ PECs using *in vitro* clonogenicity, direct differentiation analysis and *in vivo* transplantation. Based on our results, we propose that WT1^+^ PECs serve as extra-tubular progenitor cells, which are activated after severe AKI, proliferate and subsequently migrate and differentiate to mature PTECs.

## Results

### WT1 expression in PECs and PTECs is essential for renal proximal tubule repair after severe AKI

**Fig 1:**
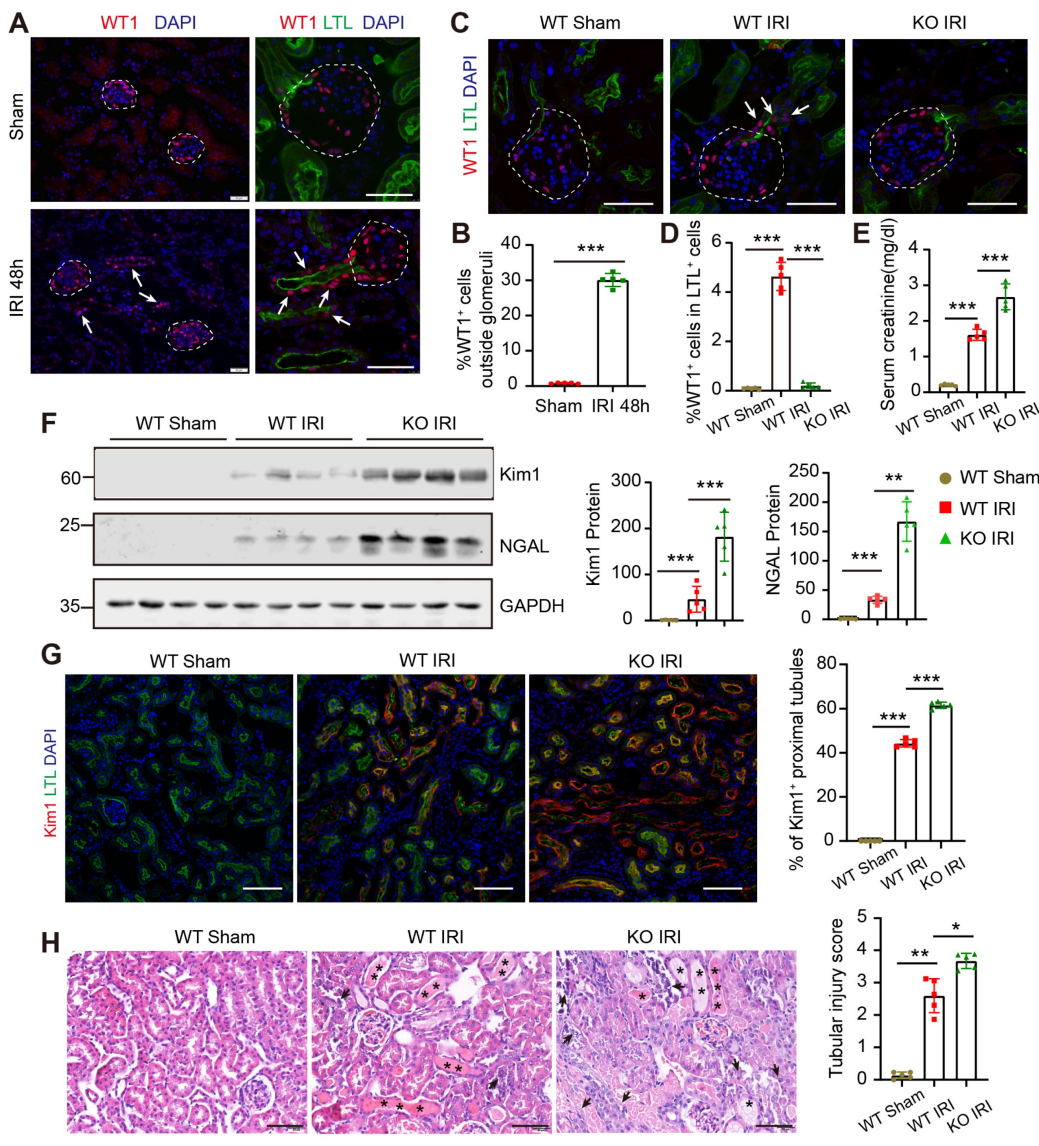
** WT1 expression in PECs and PTECs is essential for repair after severe acute kidney injury.** (A) Co-immunofluorescence staining for WT1 (red), LTL (renal proximal tubules, green), and nuclei (blue) shows increased WT1^+^ PTECs in severe injury mice compared with controls. The dotted line sketches glomeruli and arrows indicate WT1^+^ PTECs. Scalebar = 50μm. (B) Quantification of WT1^+^ cells (30.07 ± 1.87%) localized outside of the glomeruli after severe bilateral renal IRI. (C) Representative micrographs show WT1 expression in the kidney. The dotted line sketches glomeruli and arrows indicate WT1^+^ PTECs. Scalebar = 50μm. (D) Quantification of the WT1^+^ cells in LTL^+^ tubular cells. 0.09 ± 0.03% for WT Sham group, 4.64 ± 0.57% for WT IRI group, 0.21 ± 0.11% for KO IRI group. (E) Serum creatinine levels. (F) Representative Western blots showing renal expression and graphical representations of Kim1 and NGAL in each group. (G) Representative immunostaining images and quantification of Kim1 in kidney cortices. Scalebar = 100μm. (H) Representative images of H&E-stained kidney sections and quantitative scores of tubular injury 2 days post-sham or severe bilateral renal IRI. Asterisks indicate cast formation and tubular dilatation. Arrows highlight necrotic cells and cellular debris in the tubular lumen. Scalebar = 100μm. For staining quantification, 9-10 microscope fields were analyzed, and the mean data are presented as one point per mouse. * *P* < 0.05, ** *P* < 0.01, *** *P* < 0.001. n = 5 for each group of mice. PECs, parietal epithelial cells; PTECs, proximal tubular epithelial cells; IRI, ischemia-reperfusion injury; WT Sham, wild type mice (WT1^fl/fl^ mice) underwent sham surgery; WT IRI, wild type mice underwent severe bilateral renal IRI surgery 2 days after Tamoxifen administration; KO IRI, WT1 gene conditional knockout mice (Pax8^CreERT2^; WT1^fl/fl^ mice) underwent severe bilateral renal IRI surgery 2 days after Tamoxifen administration.

WT1 was expressed exclusively in the glomeruli in healthy adult kidneys. However, in a bilateral renal ischemia-reperfusion injury (IRI) induced AKI mice model, 30.07 ± 1.87% WT1^+^ cells were localized outside of the glomeruli and co-stained with Lotus tetragonolobus lectin (LTL) after severe AKI (Figure 1A-B). We also observed WT1 expression in PTECs in unilateral ureteral obstruction or cisplatin-induced AKI mice models (Figure S1A-B). WT1 expression in renal proximal tubule after AKI was rarely reported previously, and its function in PTECs after AKI was not explored.

We investigated whether the expression of WT1 in PTECs plays a protective role in kidney repair after injury, by knocking out the WT1 gene, specifically in PECs and PTECs, using a tamoxifen-inducible conditional knockout mice model. H11-Pax8-CreERT2-polyA knock-in (Pax8^CreERT2^) mice were established, as shown in Figure S2A. Pax8^CreERT2^; Rosa26- tdT^fl/+^ labeling showed that Pax8 was expressed exclusively in the kidney, and the reporter activity was observed mainly in proximal tubule and a sub-population of PECs in the Bowman's capsule (Figure S2B-D). The Pax8^+^ cells in the Bowman's capsule also expressed WT1 (Figure S2E). There was no evidence of ''leaky'' reporter activity in the kidney (Figure S2F). These results showed that the Cre activity of Pax8^CreERT2^ mice was specific to PECs and PTECs, ensuring the conditional cell specific deletion of WT1 in glomeruli PECs and renal PTECs.

Next, we generated Pax8^CreERT2^; WT1^fl/fl^ mice (WT1 knockout) and WT1^fl/fl^ mice (WT1 wild-type) by breeding WT1^fl/fl^ mice with Pax8^CreERT2^ mice. We observed that the physiological functions of podocytes and proximal tubules were not affected within 30 days after tamoxifen administration (Figure S3). Subsequently, we carried out IRI to induce severe AKI. Immunofluorescence staining showed that WT1^+^ PTECs were located ectopically along the tubular-glomerular junction at the 2nd-day post-IRI in wild-type mice, consistent with Figure 1A-B; but no WT1^+^ PTECs were found in the WT1 knockout group (Figure 1C). Quantification analysis showed that the percentage of WT1^+^ cells in LTL^+^ tubular cells was increased in the wild-type IRI group compared to the wild-type sham group, while it decreased when WT1 was specifically knocked out in the same injury condition (Figure 1D). The increased serum creatinine level indicated that WT1 deletion in PTECs and PECs exacerbated renal damage compared to the wild-type groups under the same insults (Figure 1E). The protein levels of tubular injury markers (Kim1/NGAL) were increased in WT1 knockout mice compared to wild-type mice (Figure 1F). Immunostaining with Kim1 antibody also showed more Kim1^+^ tubules in the WT1 knockout group than in the wild-type group (Figure 1G). Hematoxylin and eosin (H&E)-staining showed increased cell death in renal proximal tubules in the WT1 knockout mice, and a blinded histological scoring of tubular epithelial injury revealed an enhanced injury in the WT1 knockout mice (Figure 1H). In summary, deletion of the WT1 gene in PECs and PTECs resulted in much severe kidney injury after AKI, indicating that the expression of WT1 in PECs and PTECs is essential for the repair of proximal tubules.

### Cell lineage tracing shows the derivation of WT1^+^ PTECs from WT1^+^ PECs

**Fig 2:**
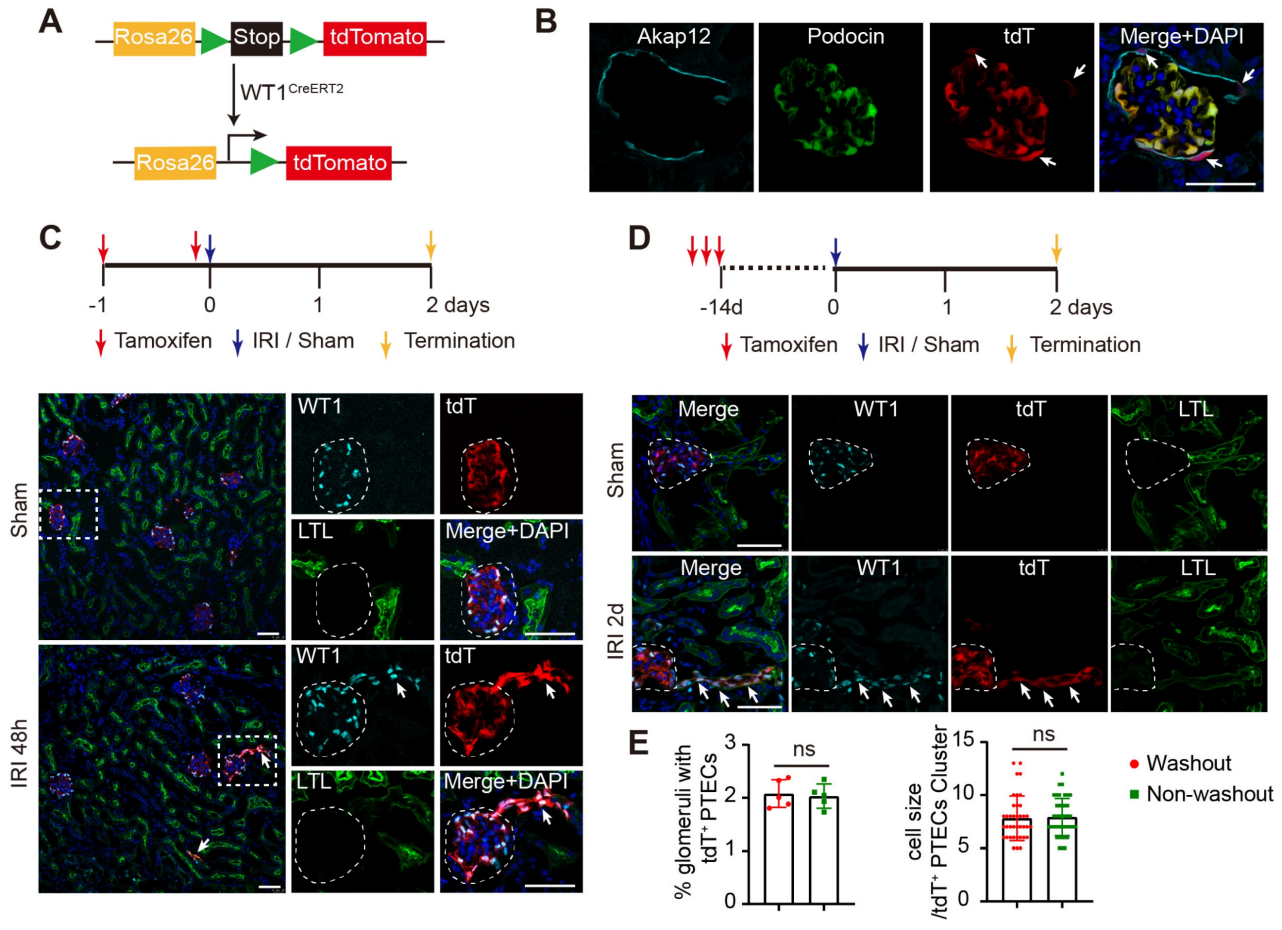
**WT1^+^ PTECs are derived partially from intrinsic WT1^+^ cells**. (A) Diagram of the generation of tamoxifen inducible WT1^CreERT2^; Rosa26-tdT^fl/+^ mice for genetic lineage tracing. (B) Immunostaining of frozen kidney sections from adult WT1^CreERT2^; Rosa26-tdT^fl/+^ mice after tamoxifen administration. Podocin, podocyte marker; Akap12, PECs marker. Arrows indicate tdT^+^ PECs. (C) “Label-injury” experimental setup. Representative micrographs show tdT^+^ cells at renal proximal tubules co-express WT1 in severe bilateral renal IRI at 48 h. (D) “Label-washout-injury” experimental setup. Representative micrographs show tdT^+^ cells at renal proximal tubules co-express WT1 in severe bilateral renal IRI at 48 h. (E) Quantification of the percentage of nephrons with tdT^+^ PTECs (2.03 ± 0.23% for non-washout; 2.08 ± 0.26% for washout; *P* = 0.764. 3 kidney slides from one mouse were analyzed and the mean data were presented as an independent point) and the number of tdT^+^ PTECs per cluster (7.93 ± 1.76 for non-washout; 7.82 ± 2.10 for washout; *P* = 0.804). ns, no significance. n = 5 biological replicates. The dotted line sketches glomeruli and arrows indicate tdT^+^ PTECs. Scalebar = 50μm. PECs, parietal epithelial cells; PTECs, proximal tubular epithelial cells; IRI, ischemia-reperfusion injury.

We performed genetic lineage tracing experiments to identify the cell origin of WT1^+^ PTECs after severe AKI. Adult mice with a cDNA encoding a Cre-modified estrogen receptor ligand-binding domain fusion protein knocked in the WT1 locus were crossed to a Cre-dependent tandem tomato (tdT) fluorescent reporter strain (Rosa26-loxp-Stop-loxp-tdT) to generate WT1^CreERT2^; Rosa26-tdT^fl/+^ mice (Figure 2A). After tamoxifen induction, the WT1^+^ reporter activity was observed in podocytes (Podocin^+^) and PECs (Akap12^+^) in the Bowman's capsule (Figure 2B). Consistent with the results displayed in Figure 1B, induction of genetic labeling of WT1^+^ cells during severe IRI and subsequent recovery led to the presence of tdT-labeled cells in some LTL^+^ tubules connecting to glomeruli, and the tdT^+^ PTECs could be stained with WT1 antibody (Figure 2C). We identified the source of the WT1^+^ PTECs following injury, by performing a multiday tamoxifen injection to maximize pre-surgery labeling of the pre-existing WT1^+^ population, and carried out two weeks of washout in WT1^CreERT2^; Rosa26-tdT^fl/+^ mice. Animals were then subjected to sham or IRI, then sacrificed after 48 h to trace the fate of the resident tdT-labeled cells. Under these conditions, irreversibly labeled tdT^+^ cells were also observed in renal proximal tubules (Figure 2D). The frequency of labeled PTECs was comparable with genetic labeling induced during IRI (2.03 ± 0.23% vs. 2.08 ± 0.26%, P = 0.764), and the number of tdT^+^ PTECs per cluster between the two groups (7.93 ± 1.76 vs. 7.82 ± 2.10, P = 0.804) was very similar (Figure 2E). These results suggested that these tdT^+^ PTECs were derived from the pre-existing resident WT1^+^ cells before the injury.

Since podocytes are terminally differentiated cells and lack of proliferation 33, we inferred that WT1^+^ PTECs might derive from the resident WT1^+^ PECs. We created a dual lineage tracing system, by introducing podocin-GFP transgenic mice, in which podocytes permanently expressed GFP, and WT1^+^ cells were labeled with tdT once treated with tamoxifen (Figure S4A). This dual lineage tracing system explicitly identified podocytes (tdT^+^GFP^+^) and WT1^+^ PECs (tdT^+^GFP^-^) in the glomeruli (Figure S4B). After severe IRI, we observed tdT^+^GFP^-^ cells in proximal tubules along the tubular-glomerular junction (Figure S4C). However, no tdT^+^ PTECs were present under the mild injury condition (Figure S4D-E). We also observed increased numbers of WT1^+^ PECs after severe injury (Figure S5A-D). Notably, the percentage of Pax8^-^WT1^+^ PECs increased from 2.9% in the normal condition to 9.4% after injury (Figure S5A-B), indicating the diversity of WT1^+^ PECs during the injury and repair process. *De novo* expression of CD44, a cell surface protein involved in cell differentiation, migration, and cell-matrix interactions, is an established marker for PEC activation 34, 35. Double staining for CD44 indicated that cells derived from WT1^ +^ PECs were activated after injury (Figure S5E). We also found that 42.28 ± 2.91% tdT^+^ PTECs co-expressed Ki67 (a proliferation marker expressed from late G1 to mitosis), and 79.91 ± 1.83% Ki67^+^ PTECs were tdT^-^ (Figure S5F). In addition, the 5-ethynyl-2'-deoxyuridine (EdU) labeling showed that 29.98 ± 1.79% of the tdT-labeled PTECs on day 3 were EdU^+^ (Figure S5G). These results suggested that the activated WT1^+^ PECs, as the extra-tubular progenitors, could proliferate and differentiate into PTECs after severe AKI.

### WT1^+^ PECs acquire transient STC phenotype and develop to mature PTECs after severe AKI

**Fig 3:**
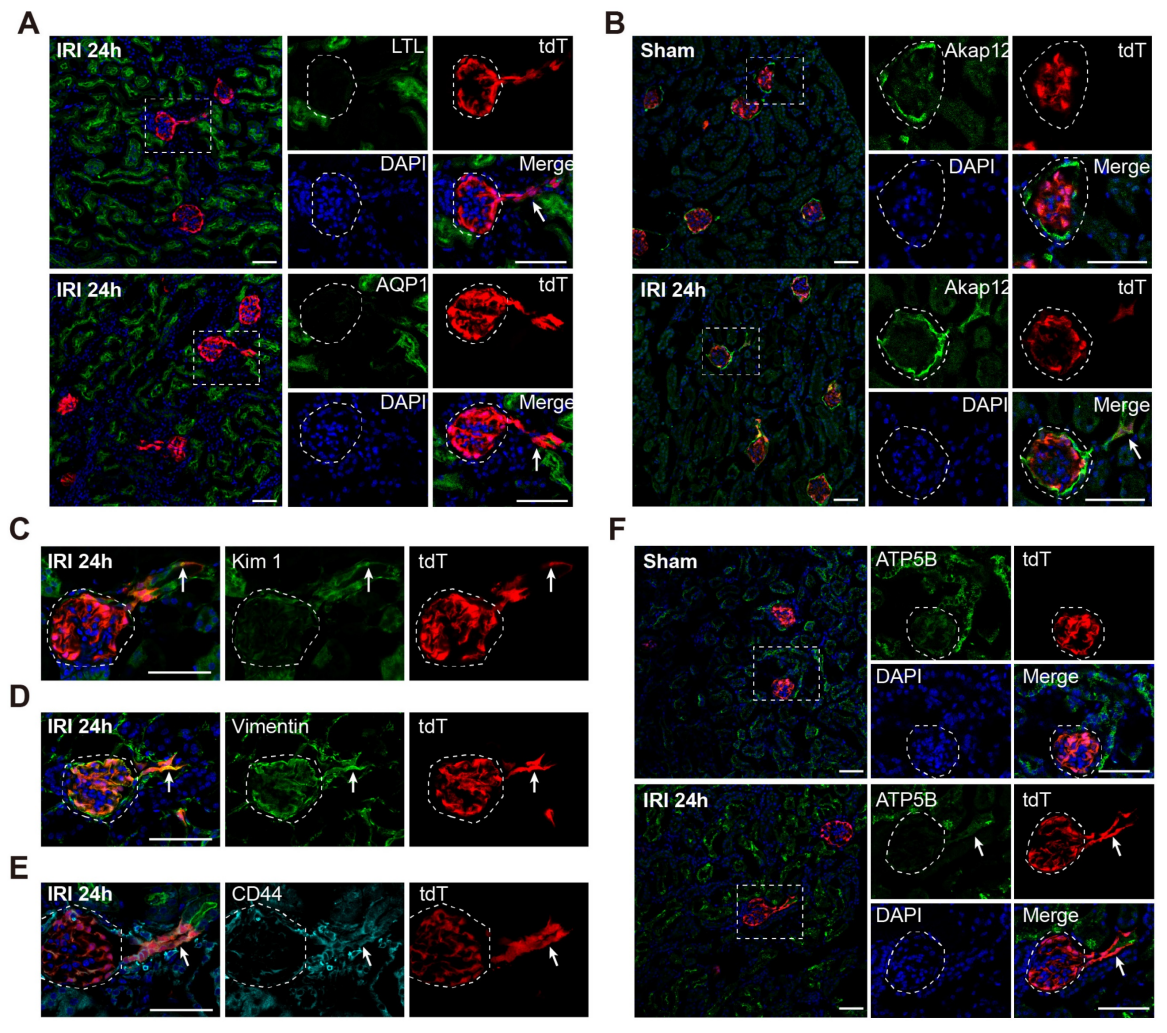
**WT1^+^ PTECs acquire transient STC phenotype in severe bilateral renal IRI at 24 h.** (A) Double staining for LTL and AQP1 shows that tdT^+^ PTECs lack expression of the brush border markers 24 h after severe IRI (arrows). (B-E) tdT^+^ PTECs share the universally acknowledged markers of STCs at 24 h after severe IRI. Double staining for Akap12(B), Kim1(C), Vimentin (D), and CD44 (E) show that most tdT^+^ PTECs co-express STCs markers (arrows). (F) Representative images of ATP5B immunostaining in the kidney 24 h after sham or severe IRI show fewer mitochondria in tdT^+^ PTECs. The dotted line sketches glomeruli. Scalebar = 50μm. PECs, parietal epithelial cells; PTECs, proximal tubular epithelial cells; STCs, scattered tubular cells; IRI, ischemia-reperfusion injury.

After demonstrating WT1^+^ PECs could develop into WT1^+^ PTECs following severe AKI, we investigated whether STCs were involved in this process. Immunostaining showed that tdT^+^ PTECs did not express STC markers 48 h after injury (Figure S6). We further examined the kidney of WT1^CreERT2^; Rosa26-tdT^fl/+^ mice sacrificed 24 h after severe IRI. Double staining for the brush border markers showed that the tdT^+^ PTECs lacked the expression of these molecules (Figure 3A). At this time, the tdT^+^ PTECs co-expressed STC markers, such as Akap12, Kim1, Vimentin, and CD44 (Figure 3B-E). We also observed that tdT^+^ PTECs harbored fewer mitochondria than the neighboring normal PTECs (Figure 3F). We did not detect STC phenotype 48 h after injury, but we observed it 24 h after injury, suggesting that the STC phenotype WT1^+^ PECs acquired during the transition into PTECs was transient.

**Fig 4:**
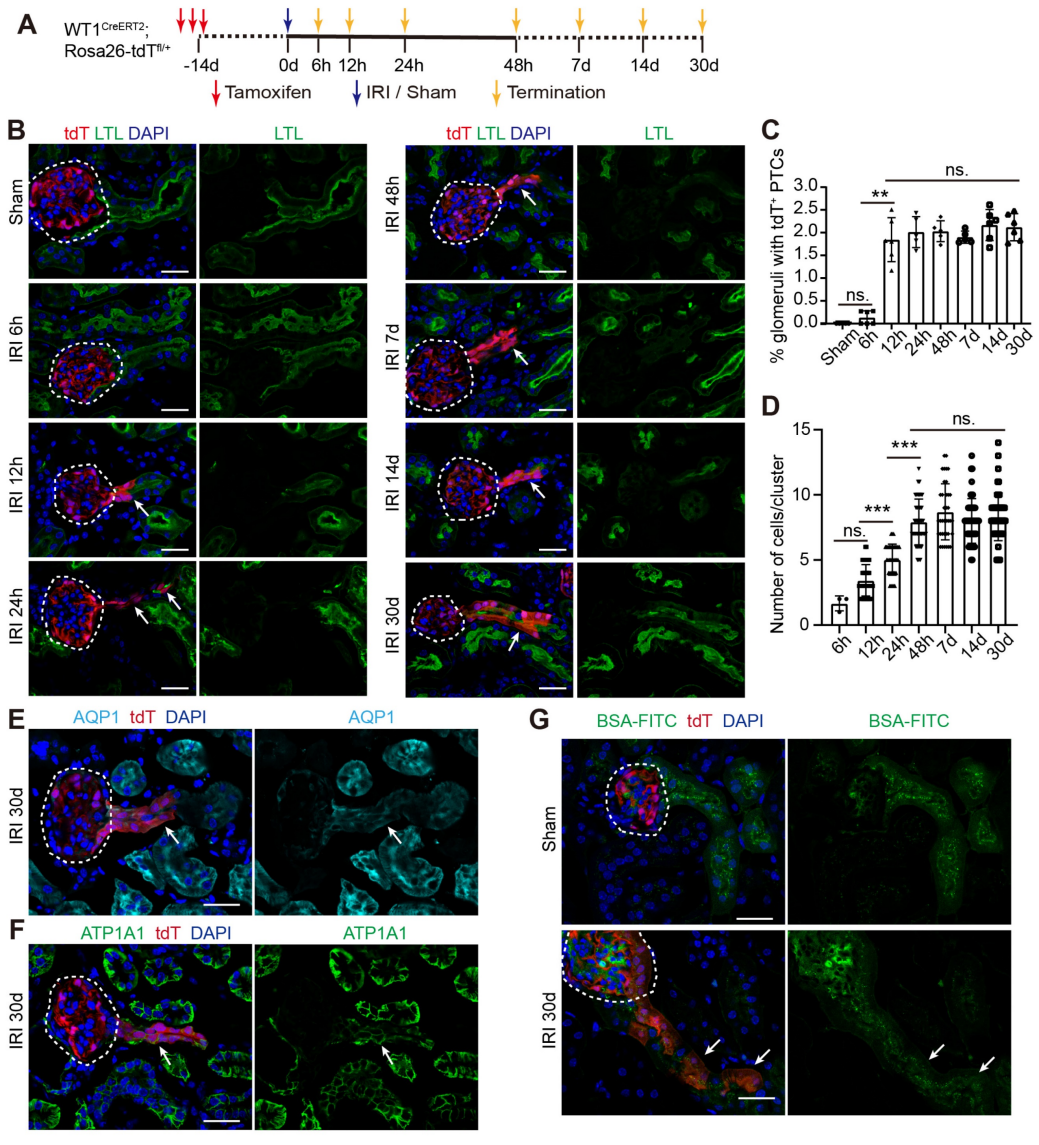
**WT1^+^ PECs develop gradually into mature PTECs after severe bilateral renal IRI.** (A) WT1^CreERT2^; Rosa26-tdT^fl/+^ mice are subjected to sham or severe IRI and sacrificed at indicated time (yellow arrows), n = 5-6 biological replicates. (B) Representative confocal micrographs show that tdT^+^ PECs migrate to the tubular-glomerular junction and acquire the brush border marker (LTL) 30 days after injury. (C, D) Quantification of the percentage of nephrons with tdT^+^ PTECs (0.13 ± 0.06% for 6 h, 1.85 ± 0.20% for 12 h, 2.01 ± 0.15% for 24 h, 2.03 ± 0.10% for 48 h, 1.90 ± 0.06% for 7 d, 2.17 ± 0.14% for 14 d, 2.12 ± 0.12% for 30 d. 3 slides from one mouse were analyzed and the mean data are presented as one point per mouse) and the number of tdT^+^ PTECs per cluster (1.67 ± 0.33 for 6 h, 3.44 ± 0.19 for 12 h, 5.03 ± 0.20 for 24 h, 7.93 ± 0.28 for 48 h, 8.70 ± 0.35 for 7 d, 8.00 ± 0.25 for 14 d, 8.32 ± 0.26 for 30 d). (E, F) Representative images of AQP1 and ATP1A1(Na-K-ATPase, located on the basolateral aspect of tubule cells) immunostaining in kidney sections at 30 days after severe IRI. (G) Representative images of BSA-FITC uptake show that the newly regenerated tdT^+^ PTECs at 30 days after the severe injury can reabsorb BSA-FITC. The dotted line sketches glomeruli and arrows indicate tdT^+^ PTECs. Scalebar = 20μm. PECs, parietal epithelial cells; PTECs, proximal tubular epithelial cells; IRI, ischemia-reperfusion injury.

Next, we recorded the detailed developmental process of WT1^+^ PECs to mature PTECs. As illustrated in Figure 4A, kidneys were analyzed at different time points after severe IRI. The kidney injury and its resolution were confirmed by histologic changes and renal function (Figure. S7A-C). Figure 4B shows that few nephrons harbored tdT^+^ PTECs in sham or 6 h post-IRI groups. However, the percentage of nephrons with tdT^+^ PTECs increased 12 h after injury (1.85 ± 0.20%) and remained constant thereafter (Figure 4B-C). The cell number of tdT^+^ PTECs per cluster increased to approximately 7.93 ± 0.28 at 48h, and then held steady (Figures 4B and 4D). We also observed that the tdT^+^ cell clusters along the tubular-glomerular junction lacked LTL signal for the first few days after injury but gradually acquired brush border markers 14 days after injury (Figures 4B and S7D) and tended to mature 30 days post-IRI (Figures 4B and 4E). By this time, the tdT^+^ PTECs expressed ATP1A1, located on the basolateral aspect of the tubular cells (Figure 4F). Furthermore, the BSA-FITC uptake assay showed the accumulation of fluorescent BSA in the tdT^+^ PTECs (Figure 4G), suggesting that the newly regenerated tdT^+^ PTECs matured enough to have the protein reabsorption function.

In conclusion, we demonstrated that WT1^+^ PECs were activated and proliferated after severe kidney insults, then migrated to the injured renal proximal tubules and acquired transient STC phenotype, ultimately developing to mature PTECs.

### scRNA-seq illustrates transcriptional features of WT1^+^ PECs

**Fig 5:**
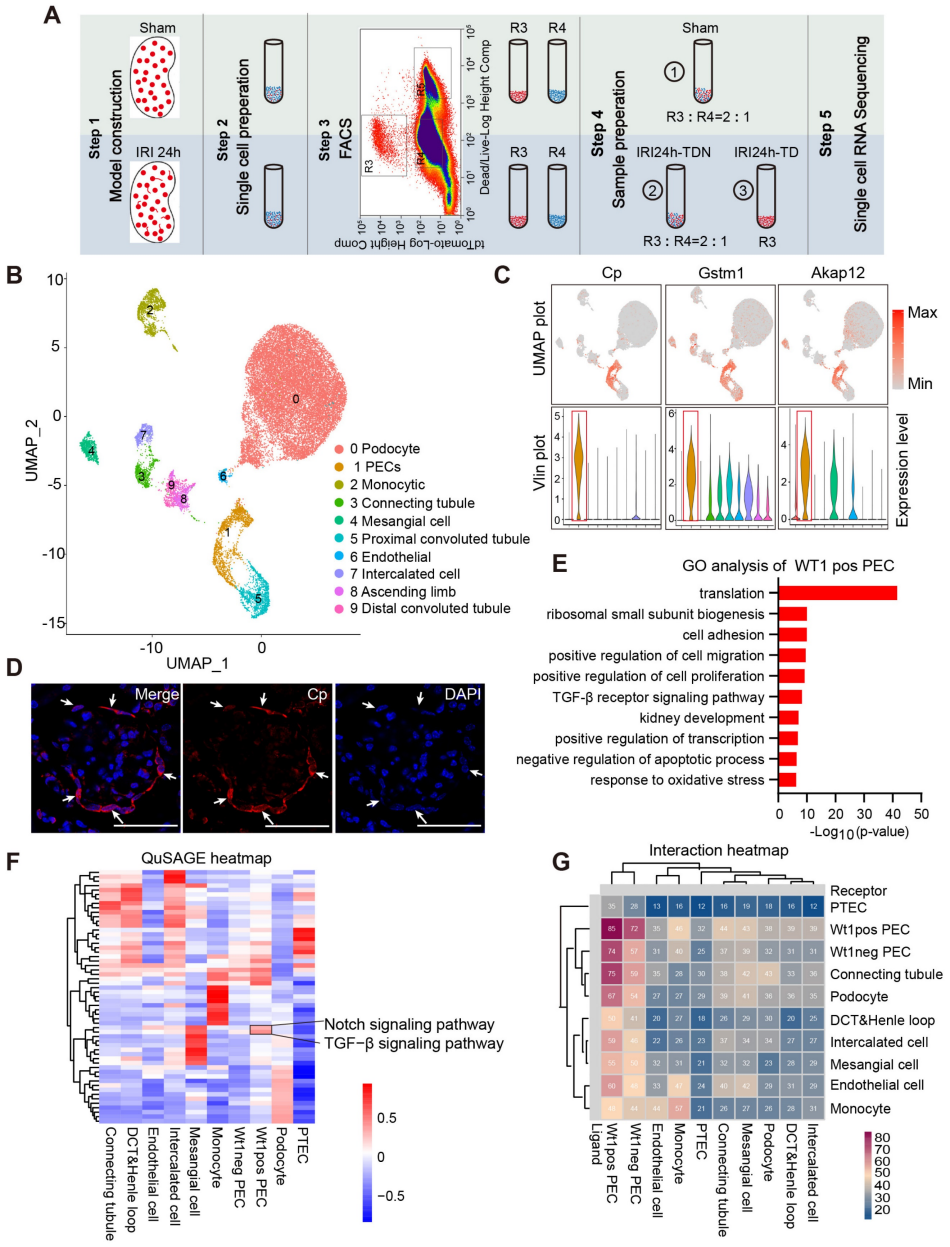
**Transcriptional features of WT1^+^ PECs**. (A) Outline of scRNA-seq experiments. Sham, single renal cell mixture from sham surgery; IRI24h-TDN, single renal cell mixture of tdT^+^ cells with tdT^-^ cells in a 2:1 ratio from mice that underwent severe bilateral renal IRI after 24 h; IRI24h-TD, single pure renal tdT^+^ cell mixture from mice that underwent severe bilateral renal IRI after 24 h. R3: tdT^+^ live cells; R4: tdT^-^ live cells; R5: dead cells. n = 4 kidneys per group. (B) UMAP plots: datasets from 3 samples integrated with Harmony. (C) Individual gene UMAP and Vlin plots show expression levels and distribution of the representative marker genes of PECs. (D) Representative micrographs of in situ hybridization of Cp. Arrows indicate Cp^+^ PECs. Scalebar = 50μm. (E) Selected GO terms of upregulated genes in the WT1^+^ PEC population. 915 genes were selected among all 22141 genes with *P* value < 5E-7. (F) Quantitative Set Analysis for Gene Expression (QuSAGE) heatmap of cell-type-enriched gene sets. (G) Interaction heatmap of all cell clusters. X-axis represents clusters as receptors and Y-axis represents clusters as ligands. The numbers indicate the number of cell-cell communication molecules between different clusters. PECs, parietal epithelial cells; PTECs, proximal tubular epithelial cells; IRI, ischemia-reperfusion injury.

We performed scRNA-seq analysis using the BD Rhapsody platform to illustrate the gene expression features of WT1^+^ PECs. As shown in Figure 5A, the single-cell suspension was prepared from the kidney cortex of WT1^CreERT2^; Rosa26-tdT^fl/+^ mice subjected to sham or severe IRI. Briefly, tdT^+^ cells were enriched by a fluorescence-activated cell sorter (FACS) to capture enough WT1^+^ PECs. TdT^+^ cells were manually mixed with tdT^-^ cells in a 2:1 ratio to get comprehensive information on the transcriptional changes of all renal cell types, particularly the healthy and damaged tubular cells. Table S1-2 shows cell viability, cell doublet rate of single-cell suspension and the parameters of cell capture. After quality control and cell filtering, 26759 cells were informative (Dataset S1) and divided into 10 clusters using the Harmony algorithm to reduce batch effects (Figure 5B). Among them, 1676 PECs were obtained, and this population displayed unique signature genes, such as Cp, Akap12 and Gstm1 (Figure 5C and Dataset S1). We validated the expression of Cp in PECs by *in situ* hybridization (Figure 5D), and the results suggested that Cp could be a novel marker gene for PECs.

Next, we focused on the transcriptional features of WT1^+^ PECs. The gene expression profile of WT1^+^ PECs is shown in Dataset S2. The GO analysis identified gene enrichment in the regulation of gene transcription, protein translation, positive regulation of cell migration, cell proliferation, and negative regulation of cell apoptosis in WT1^+^ PECs (Figure 5E). Quantitative Set Analysis for Gene Expression (QuSAGE) heatmap showed significant activation of TGF-β and the Notch signaling pathway, essential during kidney development and repair 36, 37, in WT1^+^ PECs (Figure 5F). The interaction heatmap indicated close communication of WT1^+^ PECs with other cell clusters and probably strong self-regulation in an autocrine manner (Figure 5G). These data suggested that WT1^+^ PECs might be involved in kidney regeneration after AKI.

**Fig 6:**
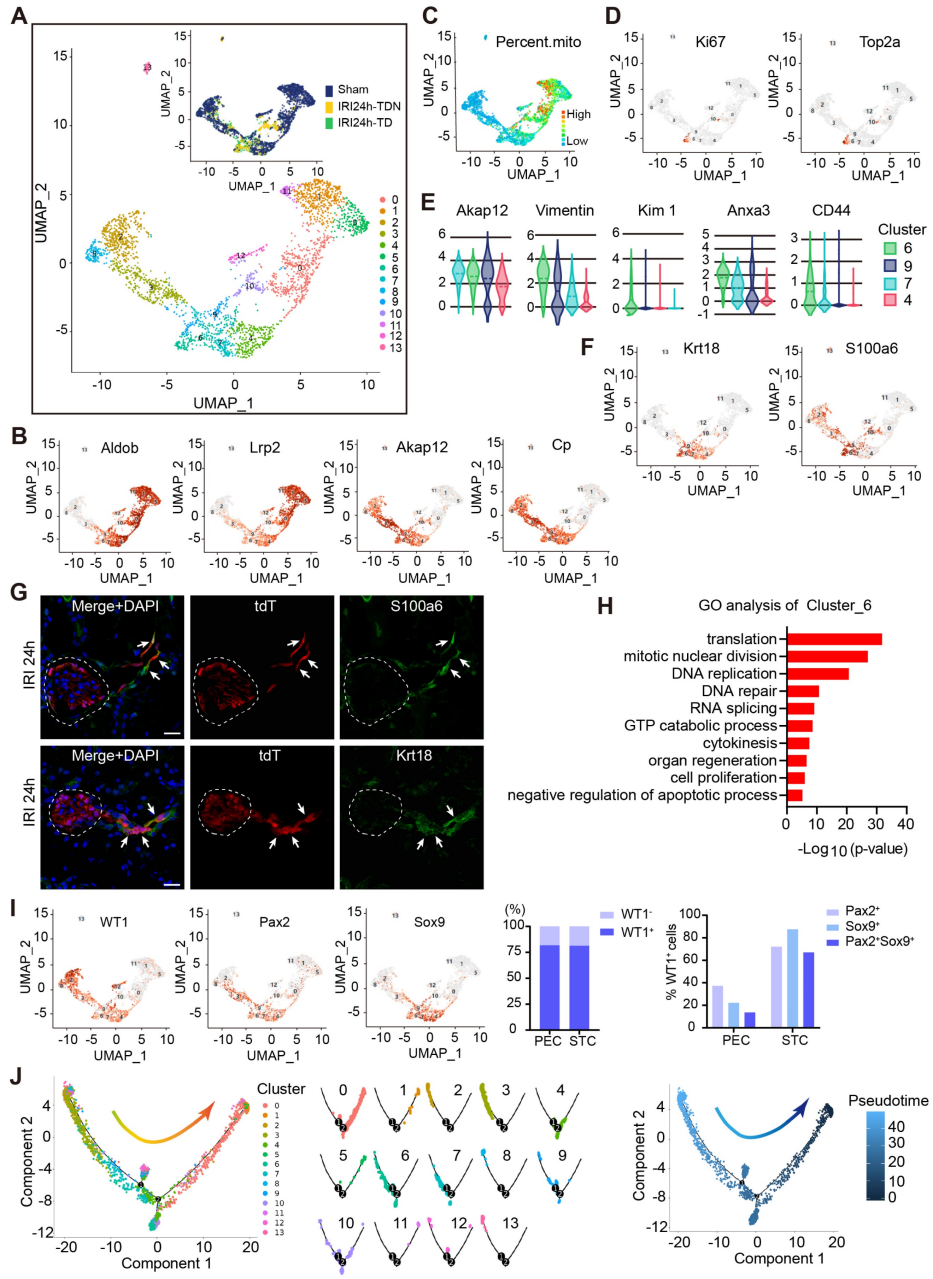
**WT1^+^ PECs serve as progenitors of PTECs and develop to mature PTECs through STC transitional stage.** (A) UMAP plots of PECs and PTECs re-clustering (2970 cells). (B) Individual gene UMAP plots show that the cluster_6/9/4/7 expressed both specific marker genes of PTECs (Aldob, Lrp2) and PECs (Akap12, Cp). (C) UMAP plots show a low percent mitochondrial gene expression in cluster_6/9/4/7. (D) UMAP plots show high expression levels of the proliferation markers (Ki67, Top2a) in cluster_6. (E) Vlin plots show high expression levels of the STC-specific marker genes in cluster_6. (F) UMAP plots of specific marker genes of cluster_6. (G) Representative immunostaining images show the expression of marker genes of cluster_6 (S100a6 and Krt18) in severe bilateral renal IRI at 24 h. The dotted line sketches glomeruli and arrows indicate tdT^+^ cells co-express S100a6 and Krt18. Scalebar = 20μm. (H) Selected GO terms of genes in cluster_6. 804 different genes were selected among all 22141 genes with *P* value < 6.47E-6. (I) UMAP plots and quantification analysis show the distribution of WT1, Pax2, and Sox9. The percentage of WT1^+^ cells in PECs (cluster_2/3/8) and STCs (cluster_6) was 81.83% and 81.31%, respectively. In WT1^+^ PECs, 37.30% was Pax2^+^, 22.21% was Sox9^+^, and 13.64% was Pax2^+^Sox9^+^; while in the WT1^+^ STCs, 72.05% is Pax2^+^, 87.58% is Sox9^+^, and 67.08% is Pax2^+^Sox9^+^. (J) Potential development trajectory of PECs and PTECs produced by Monocle 2. Arrow shows the potential developmental direction. Pseudo-time analysis in each cell cluster is shown in the middle panel. Pseudo-time (arbitrary units) is depicted from light to dark blue (right panel). PECs, parietal epithelial cells; PTECs, proximal tubular epithelial cells; STCs, scattered tubular cells; IRI, ischemia-reperfusion injury.

Furthermore, we extracted PECs and PTECs, and subclustered them into 14 cell clusters (Figure 6A). All marker genes are shown in Dataset S3. Our results illustrated that cluster_6/9/4/7 cells expressed PTECs (Aldob, Lrp2), and PECs (Akap12, Cp) markers (Figure 6B). As shown in Table S1-2, the cell multiplet rates of each sample were 4.8%, 7.1%, 5.7% respectively, and they were all excluded after cell selection. We reasoned that these cells of cluster_6/9/4/7 could not be doublets and probably represented a transitional state from PECs to PTECs. Most cells in cluster_6 came from the AKI samples (Figure 6A), suggesting that they were a novel and specific cell type after injury. Besides, cluster_6 expressed a lower level of mitochondrial genes (Figure 6C) and a higher level of proliferating genes than PTECs (Figure 6D). Among the clusters_6/9/4/7, the cluster_6 cells expressed the highest level of the STC marker genes (Figure 6E). Altogether, these results showed that cluster_6 shared the STC characteristics.

The marker genes analysis showed that cluster_6 cells mainly expressed Krt18 and S100a6 (Figure 6F and Dataset S3) validated in the tdT^+^ PTECs of 24 h post-IRI kidney by immunostaining (Figure 6G). GO analysis showed that cluster_6 cells were active in translation, mitotic division, DNA repair and organ regeneration (Figure 6H). Sox9 and Pax2 were the previously reported markers of progenitor cells involved in renal proximal tubule repair 15, 38. The percentage of WT1^+^ cells in STCs (cluster_6) and PECs (cluster_2/3/8) were not significantly different (81.31% vs 81.83%) (Figure 6I), while in WT1^+^ PECs, the percentage of cells co-expressing Sox9 or Pax2 was much lower than in WT1^+^ STCs (37.30% vs 72.05% for Pax2, 22.21% vs 87.58% for Sox9, and 13.64% vs 67.08% for co-expressing Pax2/Sox9) (Figure 6I). These data indicated that the WT1^+^ PECs were a different cell cluster from the Sox9^+^ or Pax2^+^ renal progenitors, which seemed similar to the WT1^+^ STCs in our study.

We performed computational single-cell pseudo-temporal trajectory analysis using Monocle 2 package and demonstrated a linear developmental progression from cluster_2/8/3 (PECs) to cluster_0/1/5/11 (PTECs) through the intermediate cluster_6/9/4/7 (Figure 6J). Combined with the genetic cell lineage tracing results* in vivo*, we propose that activated WT1^+^ PECs proliferated and developed into PTECs after severe IRI via STCs as the transitional stage. This process was most likely regulated by TGF-β and Notch signaling pathways.

### *In vitro* and *in vivo* differentiation of WT1^+^ PECs demonstrate their identity as a candidate for renal progenitor cells

**Fig 7:**
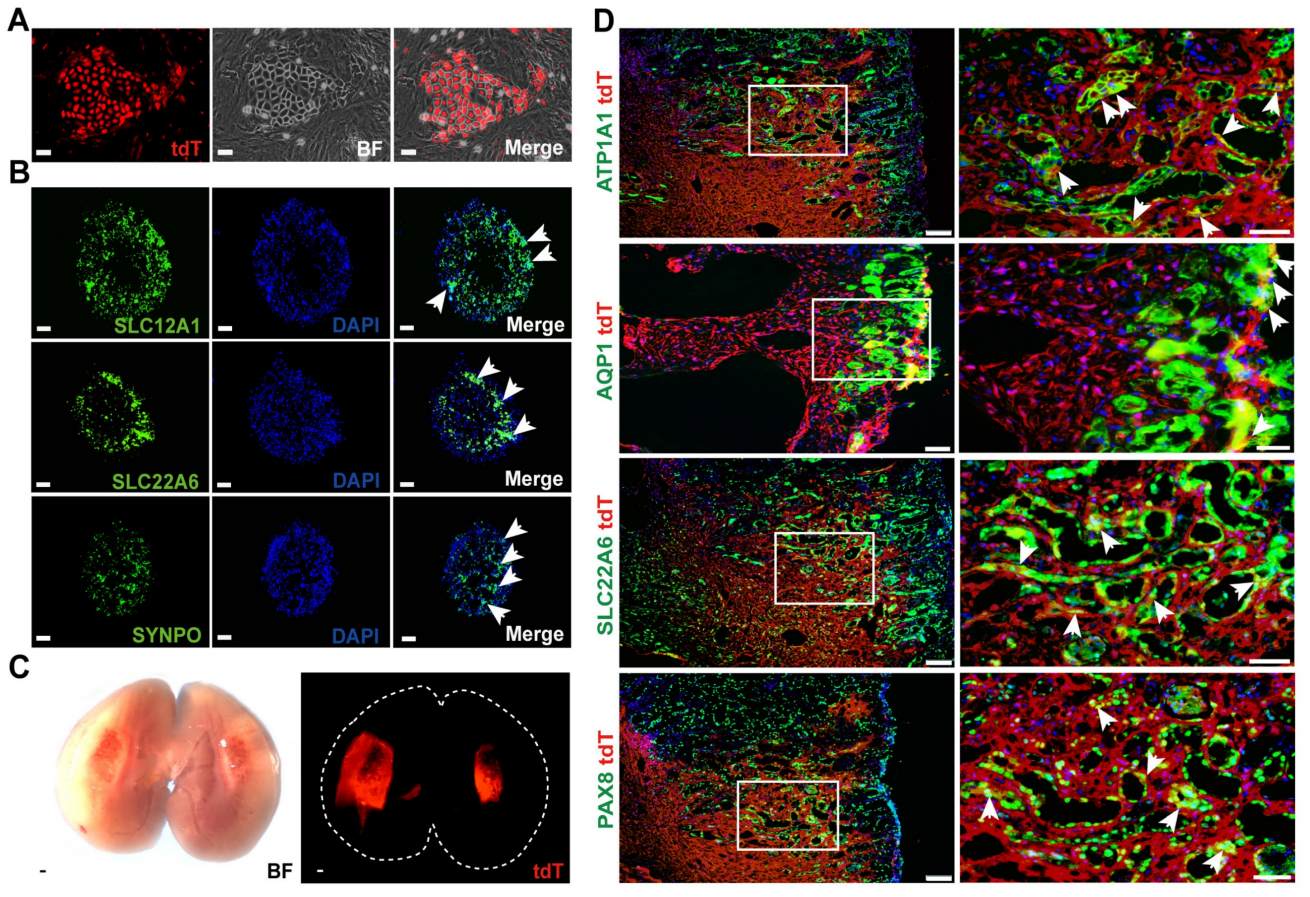
** WT1^+^ PECs show high proliferative and direct differentiation potential* in vitro* and* in vivo*.** (A) Representative images of tdT^+^ cell colonies emerging 3-5 days after seeding. Scalebar = 100μm. (B) Representative images of *in vitro* differentiation of tdT^+^ cell colonies. Arrows indicate that cell organoids after 10 days of differentiation express markers of proximal tubules (SLC22A6), thick ascending limb of loop of the Helen's (SLC12A1), and podocytes (SYNPO). Scalebar = 100μm. (C) Direct imaging of the mouse kidney following WT1^+^ cells transplantation. (D) Immuno-analysis for renal tubule markers (ATP1A1, Pax8) and proximal tubule markers (AQP1, SLC22A6) (arrows). Scalebar = 100μm (Left), 50μm (Right). PECs, parietal epithelial cells.

Tissues from the kidneys of normal adult WT1^CreERT2^; Rosa26-tdT^fl/+^ mice were dissected and digested to single cell suspensions for cell culture. We used the 3T3 feeder cell-based system to culture clonogenic cells. In this system, maintenance of parietal epithelial stem/progenitor-like cells in a ground state was supported by a combination of growth factors and regulators of TGF-β, Wnt/β-catenin, and Notch pathways, as previously reported 39. In this culture system, cell colonies usually emerged 3-5 days after seeding, grew as homogenous and round-shaped clones, as shown in Figure 7A. The proportion of tdT^+^ clones (source WT1 ^+^ PECs) was approximately 10% in all clones. The tdT^+^ clones were then manually picked for long-term culturing. They have been passaged for more than 10 generations and showed the capacity to propagate *in vitro* readily.

We used 96-well ultra-low attachment plates with a U-shaped bottom to support the 3D suspension culture of tdT^+^ cells to determine their differentiation capacity *in vitro* and the formation of kidney organoids. The cells were seeded at a density of 10^4^/well. The tdT^+^ cells spontaneously formed aggregates 24 h after seeding that were further examined to detect whether they could differentiate into mature renal tubular epithelial cells or podocytes. After 10 days of differentiation, immunostaining showed the expression of tubular epithelium markers in the human kidney organoids (Figure 7B), including SLC22A6 (S2 segment of renal proximal tubules) and SLC12A1 (thick ascending limb cells from the loop of Helen's). Some tdT^+^ clones expressed the podocyte marker synaptopodin (SYNPO). In summary, WT1^+^ PECs could be expanded and cultured into kidney organoids* in vitro*.

Next, we expanded tdT^+^ cells to 4 × 10^6^ and transplanted the cells into the incision of the injured kidneys. When transplanted kidneys were analyzed one week after transplantation, large-scale engraftment of fluorescent cells was observed (Figure 7C). Frozen sectioning and immunostaining demonstrated the incorporation of tdT^+^ cells into the incised part, which lined structures with lumens in the mouse kidney. Most cells expressed PTEC marker genes, such as AQP1 and SLC22A6, and the tubular cell marker genes ATP1A1 and Pax8 (Figure 7D). This biased differentiation fate proportion *in vivo* was consistent with the 3D organoid differentiation data presented above. In summary, our data implicated that WT1^+^ PECs could serve as renal progenitor cells and differentiate into tubular cells during the kidney regeneration/repair process after injury.

## Discussion

AKI is a major complication in hospitalized patients and is associated with high morbidity and mortality, especially in critically ill patients 40. There is a lack of effective treatments for AKI in the clinic, so innovative and effective therapies, such as regenerative medicine, need to be urgently explored. Our current study showed that activated WT1^+^ PECs could proliferate and differentiate into PTECs and replenish the lost tubular cells in severe AKI. We provided evidence that WT1^+^ PECs can engraft and differentiate into tubular cells confirming that they are extra-tubular renal progenitors.

Previously, CD24^+^CD133^+^ progenitors isolated from adult human kidneys exhibited self-renewal potential, committed toward tubular lineage, and reduced the morphologic and functional kidney damage in mice suffering from acute renal failure, suggesting that these cells can participate in tubular regeneration in adult human kidneys 11, 14, 41. However, a mouse lineage tracing experiment to validate that PECs do replace tubular cells in AKI was long awaited and is still missing. Herein, we demonstrated that WT1^+^ PECs were activated and proliferated in mice with acute renal failure and ultimately developed into mature PTECs after severe AKI. Although WT1^+^ PECs could not be assigned to any previously reported subgroup of PECs 17 based on cell shape and marker genes, our data provided evidence that this subtype of PECs served as progenitors of renal proximal tubules.

Understanding the distinct regeneration modes is significant in preventing organ injury and promoting tissue regeneration. In some organs, such as the liver 42, 43, lung 44-46, and vascular smooth muscle 47, several regeneration modes may coexist. Three different modes of renal proximal tubule regeneration have been previously proposed 48. However, whether specialized progenitors are involved in repairing injured renal proximal tubules is controversial 4-6.

Lineage tracing is a powerful tool to track the fate of cells *in vivo* and provides enhanced spatial, temporal, and kinetic resolution of the mechanisms that underlie tissue renewal and repair 49. In the kidney, the Six2-GC and SLC34a1-GFP^CreERT2^ reporter systems can label epithelial cells, including some PECs, not all PECs 4, 6. Thus, the rare WT1^+^ PECs that serve as progenitors and differentiate to PTECs in our study (approximately 2% by lineage tracing, although it could be largely underestimated in 2D visualization) are easy to be neglected in these ubiquitous lineage tracing systems. In Berger *et al.*'s study, both PECs and STCs were labeled and PEC-rtTA-positive tubular cells proliferated after IRI, but no significant increase of labeled cells was observed in the “labeling-washout-injury” experiment 5. Several possibilities may explain the discrepancy between their results and ours. First, due to the diversity of PECs, different reporter mice likely label different PEC subpopulations. Second, our study focused on the subpopulation of WT1^+^ PECs, and it has been shown that only a small subset of PTECs derived from WT1^+^ PECs. Thus, the newly regenerated PTECs we observed, if they existed in Berger *et al.*'s experiments, would probably be overlooked by using PEC-rtTA mice and the low resolution of enzymatic β-gal staining reporter system. More importantly, the severity of the injury must be taken into consideration. It has been reported that the severity of the injury determines the regenerative mechanisms and cell types involved in other organs 50-52. Also, it was proposed that intratubular progenitors might only be recruited in severe injury in the kidney 48. Consistent with this notion, in our study, WT1^+^ PECs were not activated to regenerate PTECs in the mild injury model (Figure S4D).

Rinkevich *et al* have described lineage-restricted progenitor characteristics in mammalian kidney development, maintenance, and regeneration 53. The rare PTECs derived from WT1^+^ PECs in this study could be omitted in their ubiquitous lineage tracing system. In our study, 79.91 ± 1.83% Ki67^+^ PTECs were tdT^-^(Figure S5F), indicating that survived renal proximal tubular cells participate in the repair of injured proximal tubules, as reported previously 5, 6, 53. We also demonstrated that WT1^+^ PECs contribute to renal tubule regeneration during severe renal injury. Our discovery of WT1^+^ PECs as progenitors of PTECs provides an additional regeneration route to the existing mechanisms.

There are limitations to our study. While we provided strong evidence that WT1^+^ PECs could differentiate into PTECs in severe AKI by cell lineage tracing *in vivo*, the possibility of *de novo* WT1 expression in PTECs cannot be entirely excluded since a small fraction of PTECs might not be labeled due to the low labeling efficiency. Besides, it must be conclusively shown that WT1^+^ PEC-derived PTECs are the major contributor to renal tubular repair. In the future, employing a PEC-specific CreERT2, such as Cp-CreERT2, to perform PEC-specific WT1 knockout might resolve this issue. Also, since PTECs in AKI are vulnerable to enzyme dissociation, it possible to miss some key transcriptomic information due to inadequate capture of the damaged PTECs. The outstanding key issues, including the nature of the signals that activate WT1^+^ PECs and whether WT1^+^ PECs play a similar role in human kidneys, require more research in the future.

In conclusion, in this study, we demonstrated that WT1^+^ PECs, as a type of extra-tubular progenitor-like cells, could be activated and differentiate into PTECs after severe AKI. This newly discovered mechanism of kidney repair might be a supplemental mechanism to the pre-existing renal proximal tubule regeneration modes. Elucidation of these renal progenitors may provide a potential cell origin for kidney organoids culturing and a novel strategy for renal regenerative medicine.

## Materials and Methods

### Study Approval

All animal studies were performed adhering to the Guide for the Care and Use of Laboratory Animals and approved by the Experimental Animal Committee at the Nanfang Hospital, Southern Medical University.

### Statistical Analyses

All data examined were expressed as mean ± SD. Statistical data analyses were carried out using SPSS 22.0 (SPSS Inc., Chicago, IL). Two-tailed independent sample t-tests were performed when two groups of samples were compared. Comparison between three groups was made using one-way ANOVA followed by Bonferroni test or Dunnett's T3 procedure.

Detailed materials and methods are described in Supplementary Methods.

## Supplementary Material

Supplementary methods, figures and tables, legends for datasets 1-3.

Supplementary dataset 1.

Supplementary dataset 2.

Supplementary dataset 3.

Supplementary raw images.

## Abbreviations

PECs: parietal epithelial cells
PTECs: proximal tubular epithelial cells
IRI: ischemia-reperfusion injury
STCs: scattered tubular cells
AKI: acute kidney injury
LTL: Lotus tetragonolobus lectin

## References

[1] Ronco C, Bellomo R, Kellum JA (2019). Acute kidney injury. Lancet (London, England).

[2] Scholz H, Boivin FJ, Schmidt-Ott KM, Bachmann S, Eckardt KU, Scholl UI (2021). Kidney physiology and susceptibility to acute kidney injury: implications for renoprotection. Nat Rev Nephrol.

[3] Kumar S (2018). Cellular and molecular pathways of renal repair after acute kidney injury. Kidney Int.

[4] Humphreys BD, Valerius MT, Kobayashi A, Mugford JW, Soeung S, Duffield JS (2008). Intrinsic epithelial cells repair the kidney after injury. Cell Stem Cell.

[5] Berger K, Bangen JM, Hammerich L, Liedtke C, Floege J, Smeets B (2014). Origin of regenerating tubular cells after acute kidney injury. Proceedings of the National Academy of Sciences.

[6] Kusaba T, Lalli M, Kramann R, Kobayashi A, Humphreys BD (2014). Differentiated kidney epithelial cells repair injured proximal tubule. Proc Natl Acad Sci U S A.

[7] Kumar S, Liu J, Pang P, Krautzberger AM, Reginensi A, Akiyama H (2015). Sox9 Activation Highlights a Cellular Pathway of Renal Repair in the Acutely Injured Mammalian Kidney. Cell Rep.

[8] Endo T, Nakamura J, Sato Y, Asada M, Yamada R, Takase M (2015). Exploring the origin and limitations of kidney regeneration. J Pathol.

[9] Chang-Panesso M, Kadyrov FF, Lalli M, Wu H, Ikeda S, Kefaloyianni E (2019). FOXM1 drives proximal tubule proliferation during repair from acute ischemic kidney injury. J Clin Invest.

[10] Gupta S, Verfaillie C, Chmielewski D, Kren S, Eidman K, Connaire J (2006). Isolation and characterization of kidney-derived stem cells. J Am Soc Nephrol.

[11] Sagrinati C, Netti GS, Mazzinghi B, Lazzeri E, Liotta F, Frosali F (2006). Isolation and characterization of multipotent progenitor cells from the Bowman's capsule of adult human kidneys. J Am Soc Nephrol.

[12] Langworthy M, Zhou B, de Caestecker M, Moeckel G, Baldwin HS (2009). NFATc1 identifies a population of proximal tubule cell progenitors. J Am Soc Nephrol.

[13] Lindgren D, Bostrom AK, Nilsson K, Hansson J, Sjolund J, Moller C (2011). Isolation and characterization of progenitor-like cells from human renal proximal tubules. Am J Pathol.

[14] Angelotti ML, Ronconi E, Ballerini L, Peired A, Mazzinghi B, Sagrinati C (2012). Characterization of renal progenitors committed toward tubular lineage and their regenerative potential in renal tubular injury. Stem cells (Dayton, Ohio).

[15] Kang HM, Huang S, Reidy K, Han SH, Chinga F, Susztak K (2016). Sox9-Positive Progenitor Cells Play a Key Role in Renal Tubule Epithelial Regeneration in Mice. Cell Rep.

[16] D'Agati VD, Shankland SJ (2019). Recognizing diversity in parietal epithelial cells. Kidney Int.

[17] Kuppe C, Leuchtle K, Wagner A, Kabgani N, Saritas T, Puelles VG (2019). Novel parietal epithelial cell subpopulations contribute to focal segmental glomerulosclerosis and glomerular tip lesions. Kidney Int.

[18] Appel D, Kershaw DB, Smeets B, Yuan G, Fuss A, Frye B (2009). Recruitment of podocytes from glomerular parietal epithelial cells. J Am Soc Nephrol.

[19] Berger K, Schulte K, Boor P, Kuppe C, van Kuppevelt TH, Floege J (2014). The regenerative potential of parietal epithelial cells in adult mice. J Am Soc Nephrol.

[20] Eng DG, Sunseri MW, Kaverina NV, Roeder SS, Pippin JW, Shankland SJ (2015). Glomerular parietal epithelial cells contribute to adult podocyte regeneration in experimental focal segmental glomerulosclerosis. Kidney Int.

[21] Kaverina NV, Eng DG, Freedman BS, Kutz JN, Chozinski TJ, Vaughan JC (2019). Dual lineage tracing shows that glomerular parietal epithelial cells can transdifferentiate toward the adult podocyte fate. Kidney Int.

[22] Kaverina NV, Eng DG, Miner JH, Pippin JW, Shankland SJ (2020). Parietal epithelial cell differentiation to a podocyte fate in the aged mouse kidney. Aging.

[23] Smeets B, Uhlig S, Fuss A, Mooren F, Wetzels JF, Floege J (2009). Tracing the origin of glomerular extracapillary lesions from parietal epithelial cells. J Am Soc Nephrol.

[24] Djudjaj S, Papasotiriou M, Bülow RD, Wagnerova A, Lindenmeyer MT, Cohen CD (2016). Keratins are novel markers of renal epithelial cell injury. Kidney Int.

[25] Smeets B, Kuppe C, Sicking EM, Fuss A, Jirak P, van Kuppevelt TH (2011). Parietal epithelial cells participate in the formation of sclerotic lesions in focal segmental glomerulosclerosis. J Am Soc Nephrol.

[26] Smeets B, Boor P, Dijkman H, Sharma SV, Jirak P, Mooren F (2013). Proximal tubular cells contain a phenotypically distinct, scattered cell population involved in tubular regeneration. J Pathol.

[27] Berger K, Moeller MJ (2014). Mechanisms of epithelial repair and regeneration after acute kidney injury. Semin Nephrol.

[28] Romagnani P (2011). Family portrait: renal progenitor of Bowman's capsule and its tubular brothers. Am J Pathol.

[29] Kreidberg JA, Sariola H, Loring JM, Maeda M, Pelletier J, Housman D (1993). WT-1 is required for early kidney development. Cell.

[30] Asfahani RI, Tahoun MM, Miller-Hodges EV, Bellerby J, Virasami AK, Sampson RD (2018). Activation of podocyte Notch mediates early Wt1 glomerulopathy. Kidney Int.

[31] Dong L, Pietsch S, Englert C (2015). Towards an understanding of kidney diseases associated with WT1 mutations. Kidney Int.

[32] Kaverina NV, Eng DG, Largent AD, Daehn I, Chang A, Gross KW (2017). WT1 Is Necessary for the Proliferation and Migration of Cells of Renin Lineage Following Kidney Podocyte Depletion. Stem Cell Reports.

[33] Petermann AT, Pippin J, Hiromura K, Monkawa T, Durvasula R, Couser WG (2003). Mitotic cell cycle proteins increase in podocytes despite lack of proliferation. Kidney Int.

[34] Shankland SJ, Smeets B, Pippin JW, Moeller MJ (2014). The emergence of the glomerular parietal epithelial cell. Nat Rev Nephrol.

[35] Fatima H, Moeller MJ, Smeets B, Yang HC, D'Agati VD, Alpers CE (2012). Parietal epithelial cell activation marker in early recurrence of FSGS in the transplant. Clinical journal of the American Society of Nephrology: CJASN.

[36] Mukherjee M, Fogarty E, Janga M, Surendran K (2019). Notch Signaling in Kidney Development, Maintenance, and Disease. Biomolecules.

[37] Sureshbabu A, Muhsin SA, Choi ME (2016). TGF-β signaling in the kidney: profibrotic and protective effects. Am J Physiol Renal Physiol.

[38] Lazzeri E, Angelotti ML, Peired A, Conte C, Marschner JA, Maggi L (2018). Endocycle-related tubular cell hypertrophy and progenitor proliferation recover renal function after acute kidney injury. Nat Commun.

[39] Wang X, Yamamoto Y, Wilson LH, Zhang T, Howitt BE, Farrow MA (2015). Cloning and variation of ground state intestinal stem cells. Nature.

[40] Vijayan A (2021). Tackling AKI: prevention, timing of dialysis and follow-up. Nat Rev Nephrol.

[41] Romagnani P (2009). Toward the identification of a "renopoietic system"?. Stem cells (Dayton, Ohio).

[42] Lin S, Nascimento EM, Gajera CR, Chen L, Neuhofer P, Garbuzov A (2018). Distributed hepatocytes expressing telomerase repopulate the liver in homeostasis and injury. Nature.

[43] Sun T, Pikiolek M, Orsini V, Bergling S, Holwerda S, Morelli L (2020). AXIN2(+) Pericentral Hepatocytes Have Limited Contributions to Liver Homeostasis and Regeneration. Cell Stem Cell.

[44] Chen J (2017). Origin and regulation of a lung repair kit. Nature cell biology.

[45] Kathiriya JJ, Brumwell AN, Jackson JR, Tang X, Chapman HA (2020). Distinct Airway Epithelial Stem Cells Hide among Club Cells but Mobilize to Promote Alveolar Regeneration. Cell Stem Cell.

[46] McQualter JL (2019). Endogenous lung stem cells for lung regeneration. Expert opinion on biological therapy.

[47] Majesky MW, Dong XR, Regan JN, Hoglund VJ (2011). Vascular smooth muscle progenitor cells: building and repairing blood vessels. Circulation research.

[48] Fujigaki Y (2012). Different modes of renal proximal tubule regeneration in health and disease. World J Nephrol.

[49] Romagnani P, Rinkevich Y, Dekel B (2015). The use of lineage tracing to study kidney injury and regeneration. Nat Rev Nephrol.

[50] Criscimanna A, Speicher JA, Houshmand G, Shiota C, Prasadan K, Ji B (2011). Duct cells contribute to regeneration of endocrine and acinar cells following pancreatic damage in adult mice. Gastroenterology.

[51] Tang J, Wang H, Huang X, Li F, Zhu H, Li Y (2020). Arterial Sca1(+) Vascular Stem Cells Generate De Novo Smooth Muscle for Artery Repair and Regeneration. Cell Stem Cell.

[52] Vaughan AE, Brumwell AN, Xi Y, Gotts JE, Brownfield DG, Treutlein B (2014). Lineage-negative progenitors mobilize to regenerate lung epithelium after major injury. Nature.

[53] Rinkevich Y, Montoro DT, Contreras-Trujillo H, Harari-Steinberg O, Newman AM, Tsai JM (2014). In vivo clonal analysis reveals lineage-restricted progenitor characteristics in mammalian kidney development, maintenance, and regeneration. Cell Rep.

