# Supplementary material for: WT1+ glomerular parietal epithelial progenitors promote renal proximal tubule regeneration after severe acute kidney injury
Source: Theranostics. 2023 Feb 21;13(4):1311–24. doi: 10.7150/thno.79326 (PMC10008742; doi:10.7150/thno.79326)
Supplement: Supplementary file 1 — Supplementary methods, figures and tables, legends for datasets 1-3. [file thnov13p1311s1.pdf]

Supporting Information for

WT1[+] glomerular parietal epithelial progenitors promote renal proximal tubule regeneration after severe acute kidney injury

Xizhen Hong[1,2,3,4,5,6,9#], Hao Nie[7,8#], Juan Deng[1,2,3], Shiting Liang[1,2,3],Liting Chen[1,2,3], Jing Li[1,2,3], Siqiao Gong[1,2,3], Guobao Wang[1,3,4] , Wei Zuo[7,8*], Fanfan Hou[1,2,3,4,5,6*], Fujian Zhang[1,2,3,4,5,6,10*]

[*] Fujian Zhang

Email: flykidney@163.com

This PDF file includes:

- Supplementary Methods
- Figures S1 to S7
- Tables S1 to S5
- Legends for Datasets S1 to S3
- SI References

Other supporting materials for this manuscript include the following:

- Datasets S1 to S3

1．Supplementary Methods

Animal Models

$WT1^{CreERT2/+}$ transgenic mice [1] and Rosa26-$tdTomato^{fl/+}$ mice were a kind gift from Dr. Bin Zhou and Dr. Ting Chen. $Wt1^{fl/fl}$ mice [2] were offered free by Dr. Fei Gao. Podocin-GFP transgenic mice [3] were kindly provided by Dr. Paul Epstein. B6/JGpt-H11em1Cin ($Pax8^{CreERT2}$)/Gpt mice were designed and provided by GemPharmatech Co., Ltd. Male C57BL/6 mice, weighing approximately 20-22 g, were purchased from Southern Medical University Animal Center (Guangzhou, China). Male mice above 8-week-old were used in the study and were housed in a standard environment on a regular light/dark cycle with free access to water and chow.

Renal ischemia-reperfusion injury (IRI) was established as previously described [4]. Briefly, animals were anesthetized with pentobarbital sodium (50 mg/kg body weight, intraperitoneally). Bilateral renal pedicles were clipped using microaneurysm clamps, 26 minutes for mild injury and 32 minutes for severe damage. During the ischemic period, body temperature was maintained between 37.5°C and 38°C using a temperature-controlled heating system. After removal of the clamps, reperfusion of the kidneys was visually confirmed. The sham group underwent the same procedure without clipping.

To establish unilateral ureteral obstruction model, the left ureter was exposed via a left flank incision, ligated with 4-0 silk at two points, and cut between the 2 ligation points after anesthesia. The sham operated group had no ligation.

For cisplatin nephrotoxicity, mice were given a single intraperitoneal injection of cisplatin (Sigma, P4394, USA) at 20 mg/kg body weight.

Genomic PCR

Genomic DNA was prepared from mouse tail. Tissues were lysed by incubation with proteinase K overnight at 55 °C, followed by centrifugation at 12000 g for 10 min to obtain supernatant with genomic DNA. All mice were genotyped with specific primers that distinguished the knock-in allele from the wild-type allele. The genotyping primers are listed Table S3.

Tamoxifen Administration

4-Hydroxytamoxifen (Sigma, USA) was dissolved in ethanol and corn oil at a 1:4 ratio. Tamoxifen

was introduced by intraperitoneal injection at the indicated time (0.15-0.2 mg/g body weight). The time of tamoxifen washout was referred to the previous paper [5].

Tissue Preparation and Histology

Kidneys were processed according to previously described protocols [6]. Briefly, mice were anesthetized, sacrificed, and immediately perfused via the left ventricle with ice-cold PBS. Kidneys were hemi-sectioned, and portions were snap-frozen in liquid nitrogen. Other kidneys were fixed in 4% neutral buffered formalin at 4°C for 12h, processed, embedded in paraffin wax, sectioned for the following analyses. A part of kidney was fixed in 4% PLP fixative (4% paraformaldehyde, 75mM L-lysine, 10mM sodium periodate) for 2h at 4°C, cryoprotected in 30% sucrose, and snap-frozen in optimal cutting temperature compound (OCT, ThermoFisher, USA).

Immunostaining

3-5μm sections were prepared using a cryotome (Leica microsystem, Germany) and mounted on microscope slides for immunofluorescence staining. Briefly, kidney cryosections were washed with PBST (PBS, 0.3% Triton X-100), then blocked with 5% normal donkey serum in PBST at room temperature for 1 hour. After that, slides were incubated with primary antibodies at 4°C overnight. After washing, the slides were incubated with secondary antibodies. Nuclei were stained with DAPI (Beyotime, Shanghai, China) according to the manufacturer's instructions. Images were captured by confocal fluorescence microscopy (Leica DMi8; Leica Microsystems, Buffalo Grove, IL). The antibodies are listed in Table S4.

Western Blot Analysis

Protein expression was analyzed by Western blot analysis as described previously [7]. The antibodies used are listed in Table S5.

H&E Staining and Semi-quantitative Analysis

Paraffin-embedded mouse kidney sections were prepared by routine procedure. 4μm sections were stained with H&E reagents (Solarbio, G1121, Beijing, China) according to a standard protocol.

Semi-quantitative analysis of tubular morphology was performed in a blinded fashion. Briefly, more than ten random fields (magnification, × 200) encompassing the entire cortical region per slide was assessed by quantitative measurement of tubular injury. A percentage of the area affected was estimated for the number of necrotic cells, cellular debris in the tubular lumen, cast formation,

tubular dilatation, and sloughing of the epithelial layer, and acute tubular injury score was determined as follows: 0, 0% - 5%; 1, 5% - 10%; 2, 11% - 25%; 3, 26% - 45%; 4, 46% - 75%; and 5, > 76% [8].

PAS Staining

Paraffin-embedded mouse kidney sections were prepared by routine procedure. 4μm sections were stained with PAS reagents (Baso, BA4114, China) according to a standard protocol.

EdU Proliferation Assay

For EdU proliferation assessment, mice were injected i.p. with 50 mg/kg EdU (ThermoFisher Scientific, E10187, USA) daily immediately after the injury for three times. Kidneys were dissected the next day after the last EdU injection and stained using Click-iT® Plus EdU Alexa Fluor™ 488 Imaging Kits (ThermoFisher Scientific, C10637, USA) according to the manufacturer's instructions.

BSA-FITC and Dextran Uptake Assay

For BSA-FITC uptake assay, mice were injected with 100 mg/kg BSA-FITC (Sigma, A9771, USA) through the tail vein and sacrificed 15 min after injection. For dextran uptake assay, mice were injected with 10 mg/kg Dextran, Texas Red™, 10kD (Invitrogen, D1828, USA) through the tail vein and sacrificed 1 day after injection. Dissected kidneys were fixed in 4% PLP fixative for 30 minutes at room temperature and snap-frozen in OCT. Cryosections were mounted on Fisher Superfrost Plus microscope slides for immunofluorescence examination under a confocal microscope.

Determination of Serum Creatinine and Blood Urea Nitrogen

Serum creatinine and blood urea nitrogen level was determined by an automatic chemistry analyzer (AU480; Beckman Coulter, Pasadena, CA) as described previously [9].

Preparation of Single Cell Suspension

Euthanized mice were perfused with chilled 1x PBS via the left heart. For each sample, kidneys from two $WT1^{CreERT2}$; Rosa26-$tdTomato^{fl/+}$ mice underwent sham or severe IRI were harvested, respectively. Renal cortex was minced into approximately 1 mm^3 cube and washed with DMEM/F12 (Gibco, USA) for three times. After that, tissues were digested using Multi Tissue Dissociation Kit 2 (Miltenyi. #130-110-203, Germany): 3.84 mL Buffer X, 40 µL Enzyme P, 40 µL of Buffer Y, 80 µL Enzyme D, and 16 µL Enzyme A, adding 1 mg/mL Pronase E (Roch, 459643, Switzerland), 2 mg/mL collagenase type II (Sigma , MB2665, USA). The cell suspension was

incubated for 30 mins at 37°C, then homogenized using 21G and 26 1/2G syringes. Reaction was terminated by adding 10% FBS (Gibco, USA). The solution was then passed through a 40-μm cell strainer for three times and centrifugated at 800 rpm for 5 min. The cell pellet was resuspended with 5mL DMEM/F12, incubated with 5 μL LIVE/DEAD™ Fixable Dead Cell Stain Kits (ThermoFisher, L34369, USA) on ice for 30 minutes. After that, dead cells were removed and tdT^{+} cells were enriched by FACS. Cell number and viability were analyzed using Countess AutoCounter (Invitrogen, C10227, USA). This method can generate single cell suspension with about 90% viability.

Single-cell RNA Sequencing

BD Rhapsody system was used to capture the transcriptomic information of the single cells. Single-cell capture was achieved by random distribution of a single-cell suspension across > 200000 microwells through a limited dilution approach. Beads with oligonucleotide barcodes were added to saturation so that a bead was paired with a cell in a microwell. After cell lysis, beads were collected into a single tube for reverse transcription. Upon cDNA synthesis, each cDNA molecule was tagged on the 5' end with a unique molecular identifier (UMI) and a barcode. Whole transcriptome libraries were prepared using the BD Rhapsody single-cell whole-transcriptome amplification workflow. Sequencing libraries were prepared using random priming PCR of the whole-transcriptome amplification products to enrich the 3' end of the transcripts linked with the barcode and UMI and quantified using a High Sensitivity DNA chip (Agilent, USA) on a Bioanalyzer 2200 and the Qubit High Sensitivity DNA assay (ThermoFisher, USA). The library for each sample was sequenced by Illumina sequencer (Illumina, San Diego, CA) on a 150 bp paired-end run.

Analysis of Single-cell RNA Sequencing Data

The data analysis of scRNA-seq was performed by NovelBio Co., Ltd with NovelBrain Cloud Analysis Platform. We applied fastp [10] with default parameter filtering the adaptor sequence and removed the low-quality reads to achieve the clean data. Cells contained over 200 expressed genes and mitochondria UMI rate below 50% passed the cell quality filtering and mitochondria genes were removed in the expression table. Combined datasets were integrated using the "RunHarmony" function in the Harmony package. Then PCA was constructed with top 2000 variable genes and top

10 principals were used for UMAP construction. To identify differentially expressed genes among samples, the function FindMarkers with Wilcox rank sum test algorithm was used under following criteria: 1. lnFC > 0.25; 2. p value < 0.05; 3. min.pct > 0.1.

Gene ontology (GO) analysis [11] was performed to facilitate elucidating the biological implications of marker genes and differentially expressed genes. We downloaded the GO annotations from NCBI (http://www.ncbi.nlm.nih.gov/), UniProt (http://www.uniprot.org/) and the Gene Ontology (http://www.geneontology.org/). Fisher's exact test was applied to identify the significant GO categories and FDR was used to correct the p-values.

QuSAGE analysis: The R package QuSAGE was used as described for gene set enrichment analysis to achieve the enrichment status and enrich significance of each gene sets [12].

Cell communication analysis was based on the CellPhone DB [13], a public repository of ligands, receptors and their interactions. Significant mean and Cell Communication significance (p value < 0.05) was calculated based on the interaction and the normalized cell matrix achieved by Seurat Normalization.

Pseudo-time Analysis: We applied the Single-Cell Trajectories analysis utilizing Monocle2 [14] using DDR-Tree and default parameter. Before Monocle analysis, we select marker genes of the Seurat clustering result and raw expression counts of the cells passed filtering criteria.

Fluorescence *in situ* Hybridization (FISH)

The primers for Cp probe preparation were designed according to the mRNA sequence of Mus musculus Cp from NCBI and T7 RNA polymerase promoter sequence was added to the 5' primer. The primers were synthesized by BGI Co., Ltd and the sequences were as follows:

Forward：5'- TAATACGACTCACTATAGAGGTCCTGTCATTTGGGCAG -3'

Reverse：5'- GCTGCCACATAGTAGGTCCT -3'

The FISH probe for Cp was synthesized with primers above by *in vitro* transcription and labeled with Digoxin. Kidney sections were stained with an Enhanced Sensitive ISH Detection Kit IV (Cy3) (Boster biotechnology, MK1033, China) according to a standard protocol. Briefly, cryosections of mice kidneys were washed with 0.1M PBS twice, fixed (10 min with 4% formaldehyde), and permeabilized with 70% ethanol. Before the hybridization, the cryosections were incubated with prehybridization solution at 37°C for 30 min. Then samples were hybridized with the probes for Cp

in hybridization solution at 37°C overnight. After hybridization, the sections were washed in washing buffer (2 × SSC, 0.5 × SSC, 0.2 × SSC) at 37°C for 10 min and blocked with blocking solution at 37°C for 30 min. After that, the cryosections were incubated with mouse anti-DIG antibody at 37°C for 1 hour and washed with 0.5M PBS for 4 times. Then the slides were incubated with Cy3-Donkey Anti-Mouse IgG at 37°C for 30 min. After washed with 0.5M PBS for 3 times, the samples were stained with DAPI and images were captured by a fluorescence microscopy.

Isolation and Expansion of WT1$^+$ Parietal Epithelial Cells (PECs) Labeled by tdT

The minced renal tissue, 0.2-0.5 mm^3 sizes, from WT1^{CreERT2}; Rosa26-tdTomato$^{fl/+}$ mouse, were directly digested with ice-cold buffer including DMEM/F12 (Gibco, USA), 5% FBS (Hyclone, Australia), 1% peni/strep (Life , USA), and 1 mg/mL collagenase type IV (Gibco ,USA). Then incubated at 37°C for 30-40min with gentle rocking. Dissociated cell suspensions were passed through 70-μm Nylon mesh (Falcon, USA) to remove aggregates and then washed twice with cold wash buffer (F12 medium, 5% FBS (Hyclone, Australia), 1% peni/strep (Life,USA) ,1% L-glutamine(Life, USA)). Cell viability was assessed by trypan blue dye staining. Cell pellets were collected by centrifuge at 200 g and seeded onto a feeder layer of lethally irradiated 3T3 cells in culture medium for kidney, including DMEM/F12 (Life, USA), 10% FBS (Hyclone, Australia), antibiotics, amphotericin and growth factor cocktail as previously described [15] under 37°C，7.5% CO_2 culture condition.

Non-adhered 3D Culture of WT1$^+$ PECs *in vitro*

Non-adhered WT1$^+$ PECs organoid differentiation was carried out as follows. WT1$^+$ PECs were seeded at 10^4 cells/well in 96-well U-bottom low cell-binding plates (S-bio, Japan) for organoid formation. The medium was changed to organoid differentiation medium (Advanced DMEM/F12 (Life Technologies, USA) supplemented with 1% penn/strep (Life, USA), 1X GlutaMAX (Life, USA), BSA (Sigma, USA), transferrin (PeproTech, USA), RA (Solarbio, China)). The 3D organoid structures were harvested and immunofluorescence staining was performed for further analysis of tubule and glomerulus markers followed by counterstaining with DAPI.

WT1$^+$ PECs Differentiation *in vivo* by Intra-renal Transplantation

Animal experiments were performed according to guidelines approved by the University Association for Laboratory Animal Science. Firstly, the 6-8-week C57/B6 male mouse was

anesthetized using pentobarbital sodium. Left lateral peritoneum was cut open to expose the kidney. Kidney pedicle was clamped. Then a straight incision was made on the kidney using a surgical blade. After cleaning off the blood, the kidney was sealed with medical-grade glue (Fuaile, China). TdT-labeled $WT1^{+}$ PECs were injected into the kidney. For each mouse, 4 × 10^6 cells were used. Implants formed by the $WT1^{+}$ PECs were harvested one week post transplantation and analyzed for the expression of different tubule markers.

2. Supplementary Figure Legends

Fig. S1 WT1 expresses ectopically in PTECs in severe AKI mouse models. (A) Representative WT1 immunostaining in unilateral ureteral obstruction induced severe AKI model at 48 h. (B) Representative WT1 immunostaining in cisplatin-induced severe AKI model at 48 h. The dotted line sketches glomeruli and arrows indicate $WT1^{+}$ PTECs. Scalebar = 50μm. PTECs, proximal tubular epithelial cells; AKI, acute kidney injury.

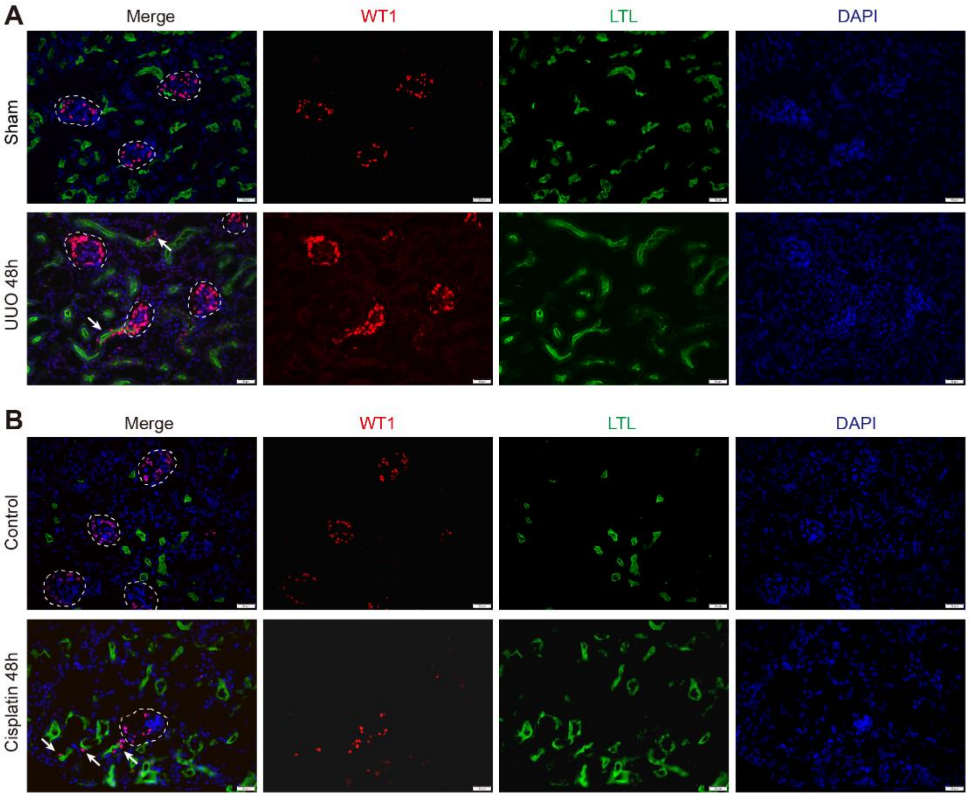

Fig. S2 The construction of $Pax8^{CreERT2}$ and its reporter activity. (A) Diagram of targeting strategy of H11-Pax8-CreERT2-polyA knock-in ($Pax8^{CreERT2}$) mouse via CRISPR/Cas9 system. (B) The expression of Pax8 in kidney, heart, liver, spleen, lung. $Pax8^{CreERT2}$ mice were crossed to

Rosa26-loxp-Stop-loxp-tdT fluorescent reporter strain, and tdT represents $Pax8^{+}$ reporter activity. Scalebar = 200μm. (C) The reporter activity of Pax8 in kidney. LTL, proximal tubules; PNA, distal tubules; DBA, collecting ducts. Scalebar = 200μm. (D) Representative images of $Pax8^{+}$ reporter activity in kidney. Akap12, parietal epithelial cell (PEC) marker. Arrows indicate $Pax8^{+}$ PECs and the dotted line sketches tubule-glomerular junction. Scalebar = 50μm. (E) Representative images of WT1 immunostaining. Arrows indicate $WT1^{+}Pax8^{+}$ PECs. Scalebar = 50μm. (F) Renal sections of $Pax8^{CreERT2}$; Rosa26-$tdT^{fl/+}$ mouse without tamoxifen administration. Scalebar = 200μm.

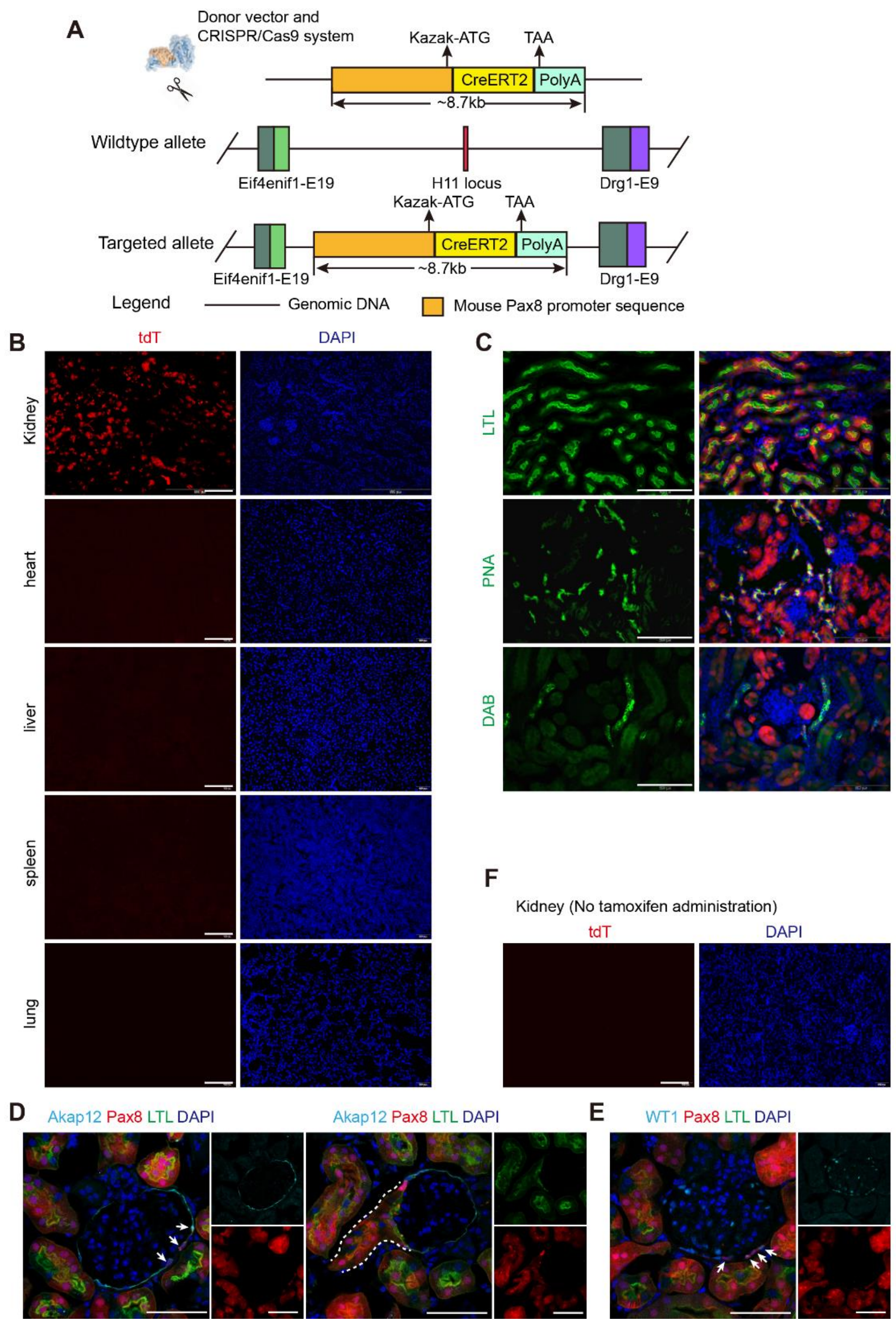

Fig. S3 WT1 conditional knockout did not affect the physiological function of the kidney. (A) Experimental setup. WT1$^{fl/fl}$ mice (wild-type, WT) and Pax8^{CreERT2}; WT1$^{fl/fl}$ mice (knockout, KO) are injected with tamoxifen, then sacrificed after 30 days, n = 5 biological replicates. (B) Representative images of PAS-stained kidney sections. Scalebar = 200μm. (C, D) Representative western blots showing the renal expression and graphical representations of WT1, SYNPO, Podocin in wild-type and WT1 knockout mice. ns, no significance. (E) Coomassie blue staining of urine

protein. An equal amount of mice urine was loaded. (F) Representative images of Dextran, Texas Red™ and BSA-FITC reabsorption of proximal tubules in wild-type and WT1 knockout mice. Scalebar = 200μm.

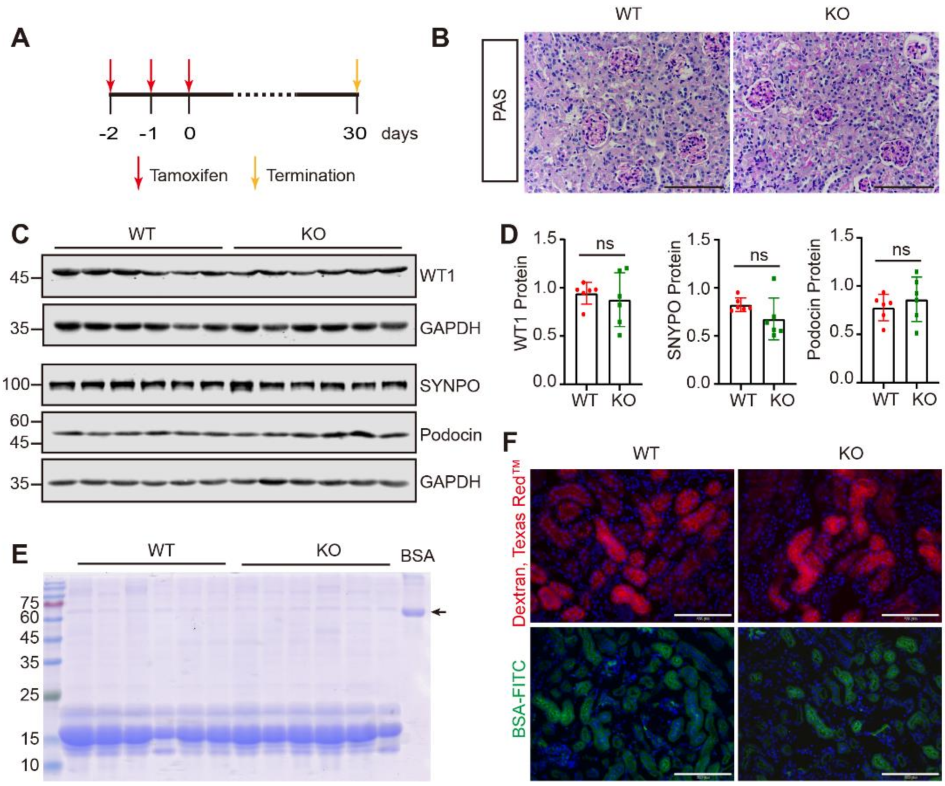

Fig. S4 Dual lineage tracing system shows tdT$^+$GFP$^-$ cells in renal proximal tubule in severe ischemia-reperfusion injury (IRI), but detects no tdT$^+$ proximal tubular epithelial cells (PTECs) in mild IRI model. (A) Schematic diagram of the generation of WT1^{CreERT2}; Rosa26-tdTomato$^{fl/+}$; podocin-GFP (WT1-PODO) mice. (B) Frozen kidney sections show WT1$^+$ cells are labeled with tdT and podocytes are permanently labeled with GFP. Rare tdT$^+$GFP$^-$ cells (WT1$^+$ PECs) along the glomerular tuft are clearly recognized (Arrows). Scale bar = 100μm. (C) Representative images of kidney sections of WT1-PODO mice 48 h after sham or severe IRI. GFP labels podocytes and tdT represents WT1$^+$ cells. Arrowheads indicate tdT$^+$ GFP$^-$ cells along the glomerular tuft. Arrows indicate tdT$^+$ GFP$^-$ cells along the tubule-glomerular junction. Scalebar = 150μm. (D) Representative micrographs and quantification show no tdT-labeled WT1 expression in tubules in sham or mild injury mice models, but the percentage of nephrons with tdT$^+$ PTECs increases (2.04 ± 0.14%) after severe IRI. 3 slides from one mouse were analyzed and the mean data was presented as one point per mouse. Scalebar = 100μm. (E) Serum creatinine analysis. * $P < 0.05$, *** $P < 0.001$. n = 5 biological replicates.

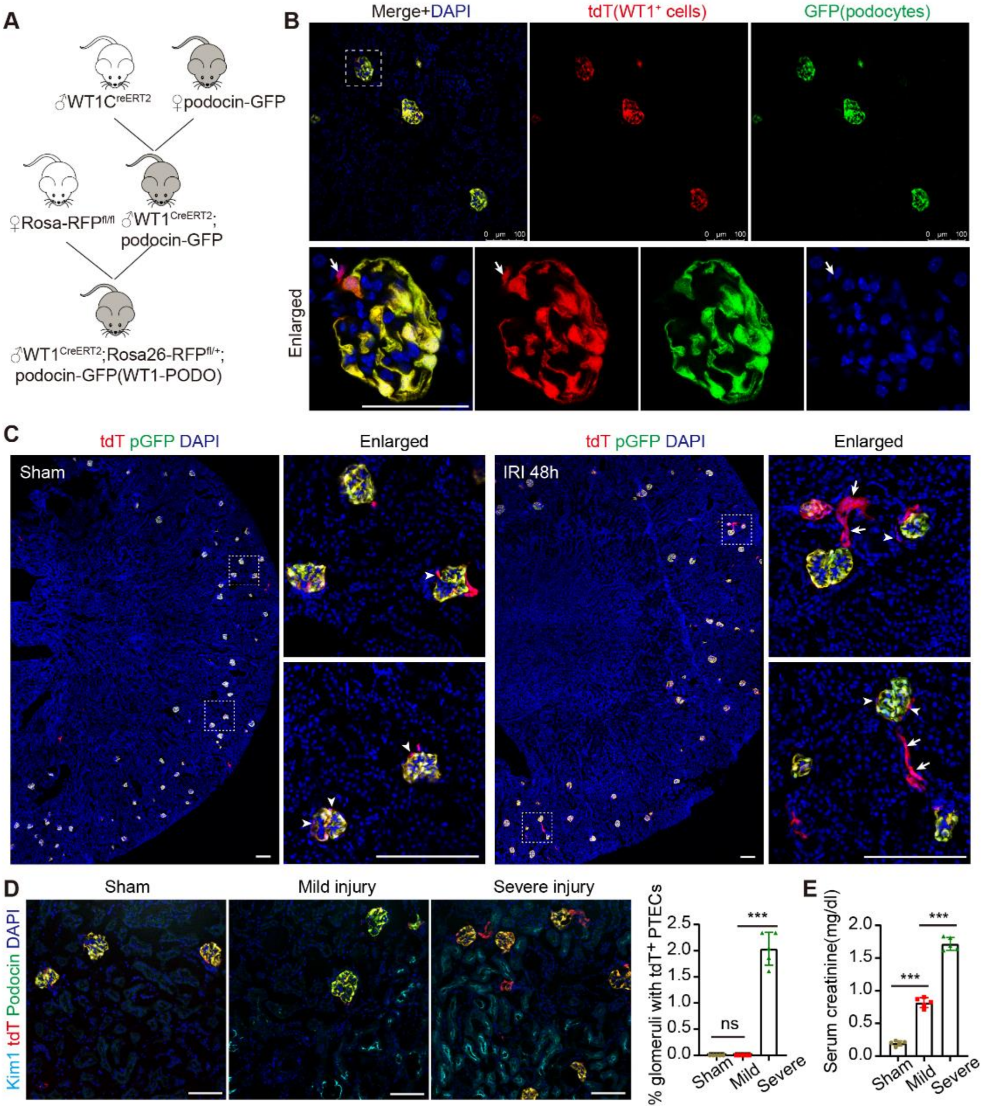

Fig. S5 The newly generated tdT-labeled PTECs are derived from activated WT1$^+$ PECs. (A) Double staining for Pax8, the marker of PECs. Arrows indicate Pax8$^+$ tdT$^+$ PECs subpopulation and arrowheads indicate Pax8$^-$ tdT$^+$ PECs subpopulation. Scalebar = 50μm. (B) Quantification of the number of tdT$^+$ PECs per glomerulus (3.10 ± 0.08 *vs.* 1.34 ± 0.06 for Pax8$^+$ tdT$^+$ PECs, 0.32 ± 0.04 *vs.* 0.04 ± 0.02 for Pax8$^-$ tdT$^+$ PECs). *** $P < 0.001$. (C) Double staining for Akap12, PEC marker. Arrows indicate Akap12$^+$ tdT$^+$ PECs subpopulation. Scalebar = 50μm. (D) Quantification of the number of tdT$^+$ PECs per 100μm glomerular tuft (1.26 ± 0.02 *vs.* 0.44 ± 0.02/100μm). *** $P < 0.001$. (E) Double staining for CD44, the marker of activated PECs. Arrows denote activated tdT$^+$ PECs. Scalebar = 50μm. (F) Immunostaining for Ki67. Arrows indicate Ki67$^+$ tdT$^+$ PTECs and arrowheads indicate Ki67$^+$ tdT$^-$ PTECs. The dotted line sketches glomeruli. Scalebar = 50μm. Quantification analysis shows 42.28 ± 2.91% tdT$^+$ PTECs co-expressed Ki67, and 79.91 ± 1.83% Ki67$^+$ PTECs

were tdT$^-$. At least 7 microscope fields were analyzed and the mean data were presented as one point per mouse. n = 5 biological replicates. (G) Representative images of EdU staining. Scalebar = 50μm. Arrows indicate EdU$^+$ tdT$^+$ cells. Quantification analysis shows 29.98 ± 0.80% tdT$^+$ cells are EdU$^+$. At least 7 microscope fields were analyzed and the mean data were presented as one independent point. n = 5 biological replicates. PECs, parietal epithelial cells; PTECs, proximal tubular epithelial cells.

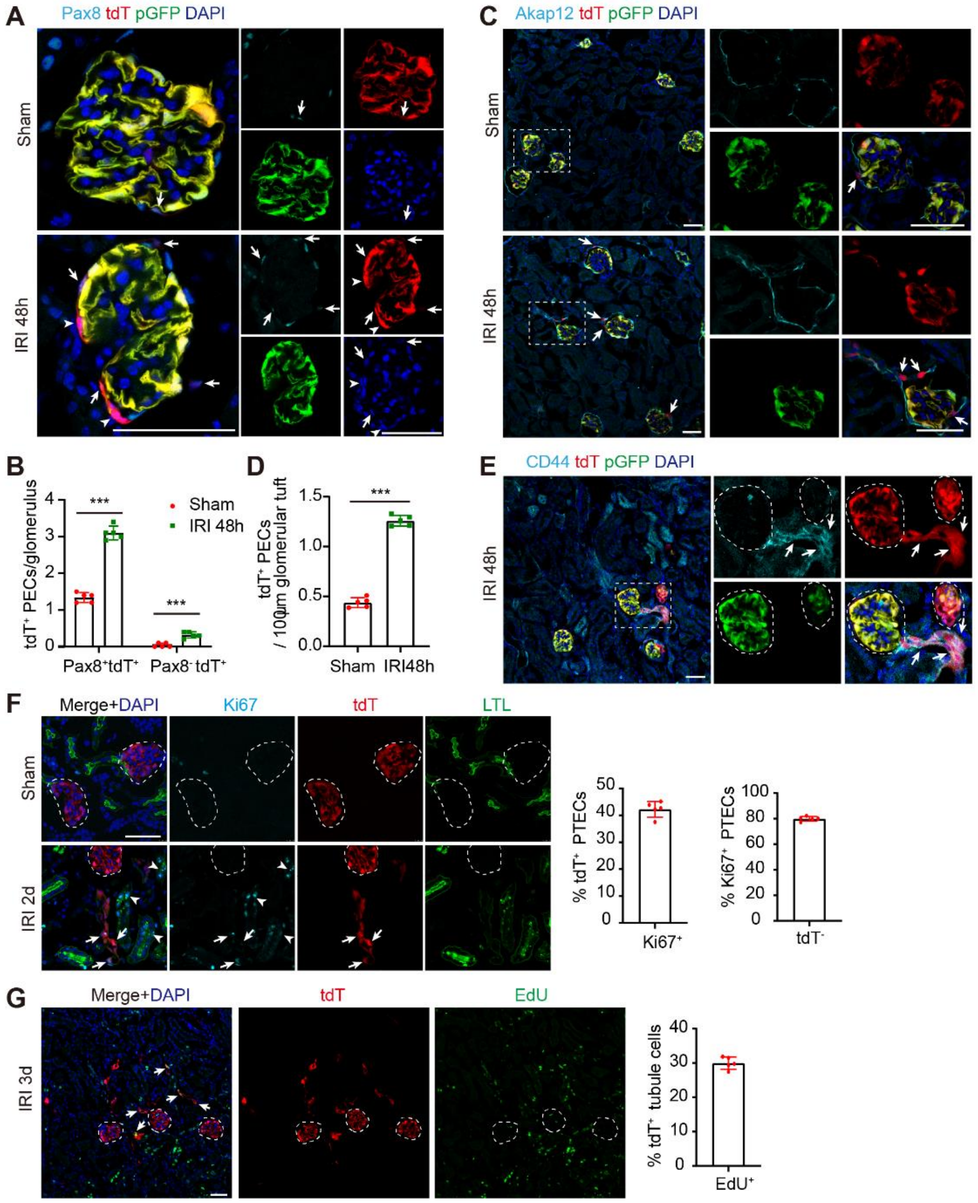

Fig. S6 tdT$^+$ proximal tubular epithelial cells (PTECs) share no scattered tubular cell (STC) markers in IRI-induced severe acute kidney injury model at 48 h. (A, B) Representative micrographs show that tdT$^+$ PTECs do not express the STC markers Kim1(A) and Vimentin (B) at 48 h post IRI. Scalebar = 75μm. IRI, ischemia-reperfusion injury.

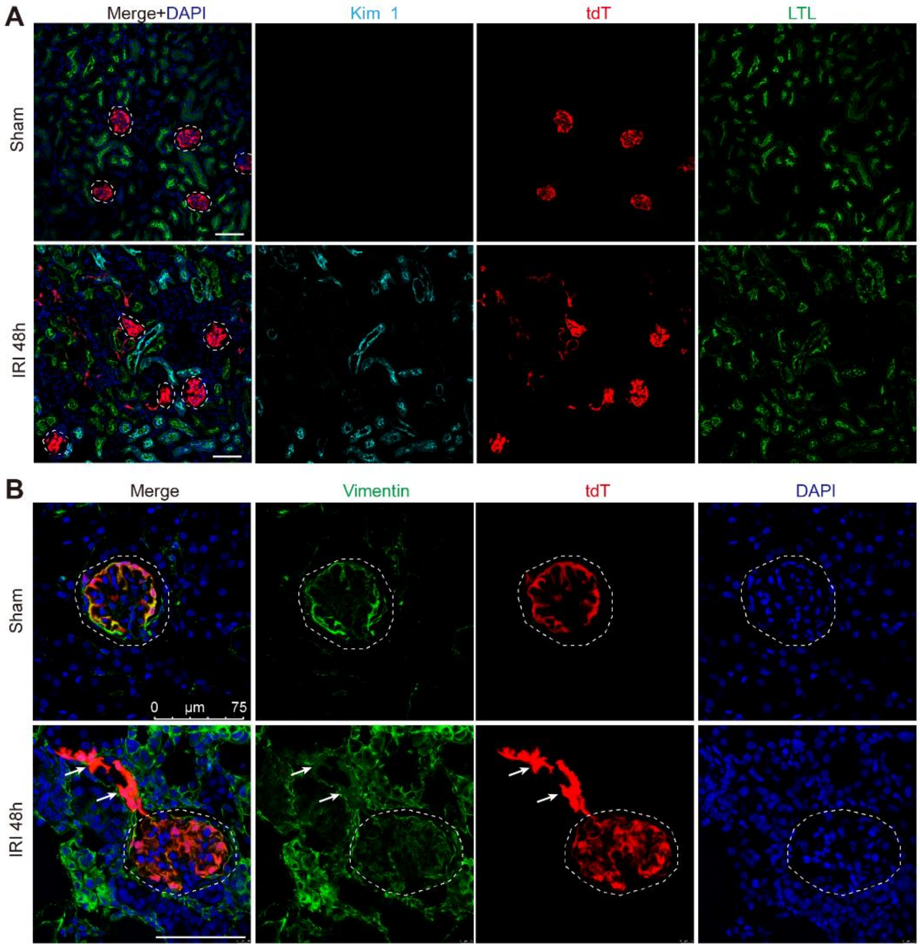

Fig. S7 Kidney injury assessment during 30 days after severe IRI. (A) Representative images of H&E staining. Scalebar = 200μm. (B, C) Kidney function assessment. Quantification of serum creatinine (B) and blood urea nitrogen (C) levels. (D) Immunostaining for AQP1 in kidney sections of 14 days after IRI. Scalebar = 20μm. IRI, ischemia-reperfusion injury.

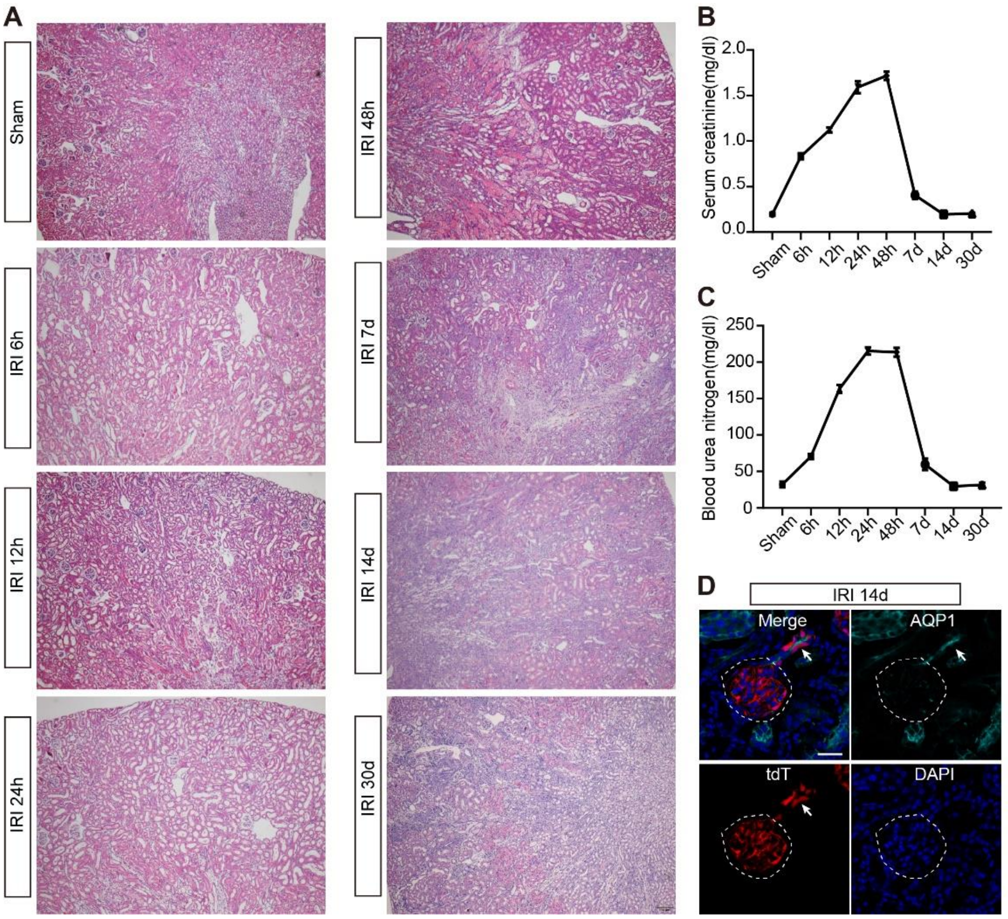

3. Supplementary Tables

Table S1: The cell viability and cell doublet rate of single cell suspension

Sample	Sham	IRI24h-TD	IRI24h-TDN
cell viability	88.06%	95.62%	92.59%
cell doublet rate	5.20%	5.20%	5.20%

Table S2: The parameters of cell capture

Sample	Sham	IRI24h-TD	IRI24h-TDN
Number of wells with viable cells at cell load	14094	16837	15777
Number of viable cells captured in wells at cell load	14969	18114	16846
Cell multiplet rate at cell load	5.8%	7.1%	6.2%
Number of wells with viable cells and a bead	12444	16005	14803
Number of viable cells captured in wells with a bead	13088	17222	15714
Cell multiplet rate	4.8%	7.1%	5.7%

Table S3. Primer sequences used in genotyping.

Gene	Primer Sequence
WT1-Cre (Mutant: 286bp, WT: Negative)	Forward: GCATAACCAGTGAAACAGCATTGCTG
	Reverse: GGACATGTTCAGGGATCGCCAGGCG
Rosa26 (Mutant:196bp, WT:297bp)	Pr1590: AAGGGAGCTGCAGTGGAGTA
	Pr1591: CCGAAAATCTGTGGGAAGTC
	Pr1592: GGCATTAAAGCAGCGTATCC
	Pr1593: CTGTTCCTGTACGGCATGG
$WT1^{fl/fl}$ (Mutant: 210bp, WT: 150bp)	Forward: TGCCTACCCAATGCTCATTG
	Reverse: GAAACTGTTTGTAACGAGAG
Pax8-Cre (Mutant: 121bp, WT: Negative)	Forward: CTATTGGACTTTGACTGCAGGGGC
	Reverse: CTGATCCACAGCCAGGTTTTGC

Table S4. Antibodies for immunostaining.

Antibody	Source	Cat#	Dilution ratio

Akap12	Proteintech	25199-1-AP	1:100
AQP1	Proteintech	20333-1-AP	1:100
ATP1A1	Proteintech	14418-1-AP	1:100
ATP5B	Proteintech	17247-1-AP	1:100
CD44	Boster biotechnology	A00052	1:200
Ki67	Genetex	GTX16667	1:500
Kim1	Boster biotechnology	BA3537	1:100
Krt18	Boster biotechnology	A01357-1	1:100
NPHS2	Abcam	ab181143	1:200
Pax8	Abcam	ab189249	1:200
S100A6	Proteintech	10245-1-AP	1:100
SLC22A6	Abcam	ab135924	1:200
SYNPO	Proteintech	21064-1-AP	1:200
Vimentin	Abcam	ab92547	1:100
WT1	Abcam	ab89901	1:50
SLC12A1	Proteintech	18970-1-AP	1:200
Cy5-AffiniPure Donkey Anti-Rabbit IgG	Jackson ImmunoResearch	711-175-152	1:200
Goat Anti-Rabbit IgG H&L (Alexa Fluor® 488)	Abcam	ab150077	1:500
Cy3-AffiniPure Donkey Anti-Rabbit IgG	Jackson ImmunoResearch	711-165-152	1:200

Table S5. Antibodies for western blot.

Antibody	Source	Cat#	Dilution ratio
NPHS2	Abcam	ab181143	1:2000
SYNPO	Proteintech	21064-1-AP	1:1000
WT1	Proteintech	12609-1-AP	1:1000
Kim1	R&D Systems	AF1817	1:2000
NGAL	Abcam	ab63929	1:1000
GAPDH	Affinity	T0004	1:3000
goat anti-mouse IRDye 800CW	LI-COR	926-32210	1:20000
goat anti-rabbit IRDye 800CW	LI-COR	926-32211	1:20000
Donkey anti-Goat IRDye 800CW	LI-COR	926-32214	1:20000

Reference

1. Zhou B, Ma Q, Rajagopal S, Wu SM, Domian I, Rivera-Feliciano J, et al. Epicardial progenitors contribute to the cardiomyocyte lineage in the developing heart. Nature. 2008; 454: 109-13.
2. Gao F, Maiti S, Alam N, Zhang Z, Deng JM, Behringer RR, et al. The Wilms tumor gene, Wt1, is required for Sox9 expression and maintenance of tubular architecture in the developing testis. Proc Natl Acad Sci U S A. 2006; 103: 11987-92.
3. Xu J, Zheng S, Kralik PM, Krishnan L, Huang H, Hoying JB, et al. Diabetes Induced Changes in Podocyte Morphology and Gene Expression Evaluated Using GFP Transgenic Podocytes. Int J Biol Sci. 2016; 12: 210-8.
4. Wei Q, Dong Z. Mouse model of ischemic acute kidney injury: technical notes and tricks. Am J Physiol Renal Physiol. 2012; 303: F1487-94.
5. Sun T, Pikiolek M, Orsini V, Bergling S, Holwerda S, Morelli L, et al. AXIN2(+) Pericentral Hepatocytes Have Limited Contributions to Liver Homeostasis and Regeneration. Cell Stem Cell. 2020; 26: 97-107 e6.
6. Humphreys BD, Valerius MT, Kobayashi A, Mugford JW, Soeung S, Duffield JS, et al. Intrinsic epithelial cells repair the kidney after injury. Cell Stem Cell. 2008; 2: 284-91.
7. Feng X, Hong X, Fan Q, Chen L, Li J, Deng J, et al. dCubilin- or dAMN-mediated protein reabsorption in Drosophila nephrocytes modulates longevity. Disease models & mechanisms. 2021; 14.
8. Kumar S, Liu J, Pang P, Krautzberger AM, Reginensi A, Akiyama H, et al. Sox9 Activation Highlights a Cellular Pathway of Renal Repair in the Acutely Injured Mammalian Kidney. Cell Rep. 2015; 12: 1325-38.
9. Luo C, Zhou S, Zhou Z, Liu Y, Yang L, Liu J, et al. Wnt9a Promotes Renal Fibrosis by Accelerating

Cellular Senescence in Tubular Epithelial Cells. J Am Soc Nephrol. 2018; 29: 1238-56.
10. Chen S, Zhou Y, Chen Y, Gu J. fastp: an ultra-fast all-in-one FASTQ preprocessor. Bioinformatics (Oxford, England). 2018; 34: i884-i90.
11. Ashburner M, Ball CA, Blake JA, Botstein D, Butler H, Cherry JM, et al. Gene ontology: tool for the unification of biology. The Gene Ontology Consortium. Nat Genet. 2000; 25: 25-9.
12. Yaari G, Bolen CR, Thakar J, Kleinstein SH. Quantitative set analysis for gene expression: a method to quantify gene set differential expression including gene-gene correlations. Nucleic acids research. 2013; 41: e170.
13. Vento-Tormo R, Efremova M, Botting RA, Turco MY, Vento-Tormo M, Meyer KB, et al. Single-cell reconstruction of the early maternal-fetal interface in humans. Nature. 2018; 563: 347-53.
14. Trapnell C, Cacchiarelli D, Grimsby J, Pokharel P, Li S, Morse M, et al. The dynamics and regulators of cell fate decisions are revealed by pseudotemporal ordering of single cells. Nature biotechnology. 2014; 32: 381-6.
15. Zuo W, Zhang T, Wu DZ, Guan SP, Liew AA, Yamamoto Y, et al. p63(+)Krt5(+) distal airway stem cells are essential for lung regeneration. Nature. 2015; 517: 616-20.
