# Supplementary figures and images for: WT1+ glomerular parietal epithelial progenitors promote renal proximal tubule regeneration after severe acute kidney injury
Source: Theranostics. 2023 Feb 21;13(4):1311–24. doi: 10.7150/thno.79326 (PMC10008742; doi:10.7150/thno.79326)

**Fig. 1F**

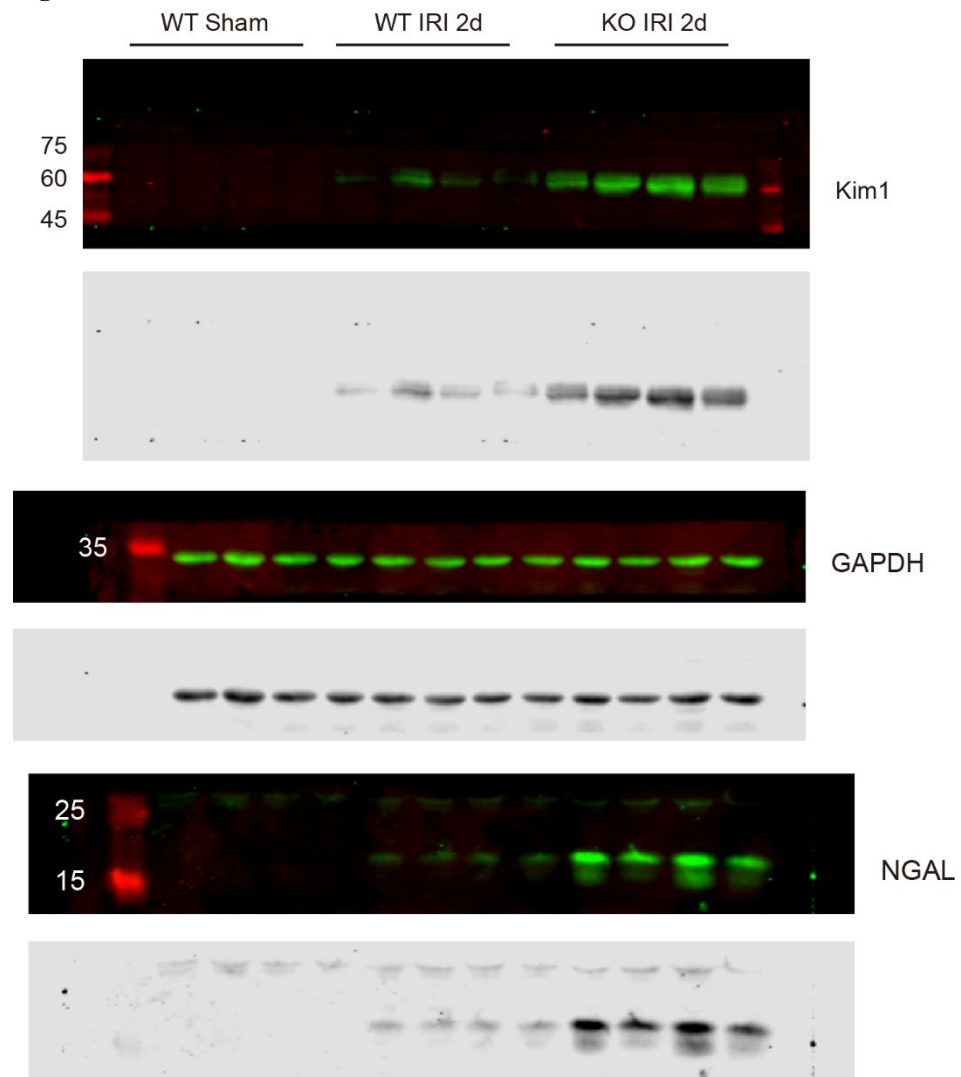

Fig. S3C

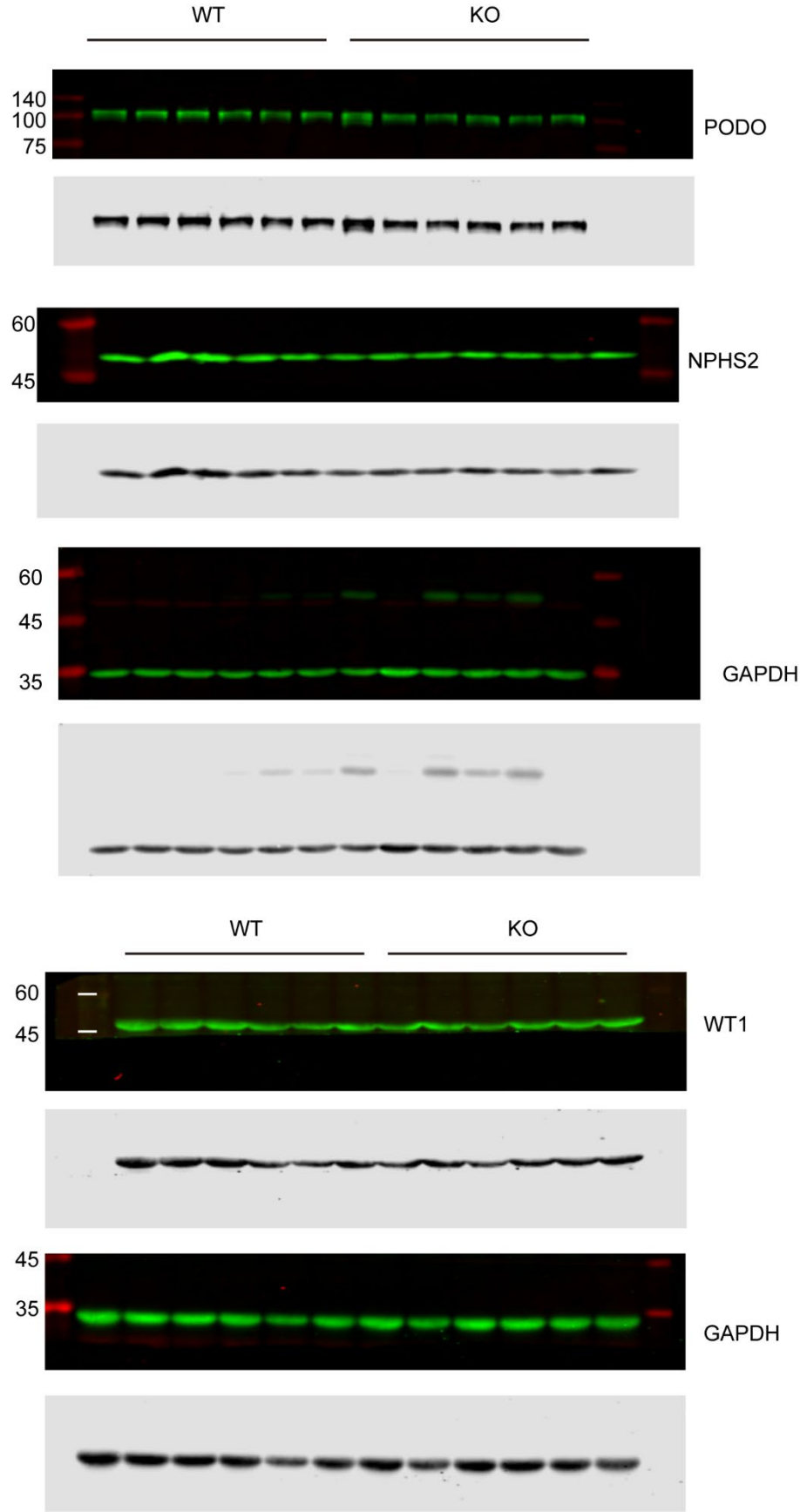

Fig. 1G

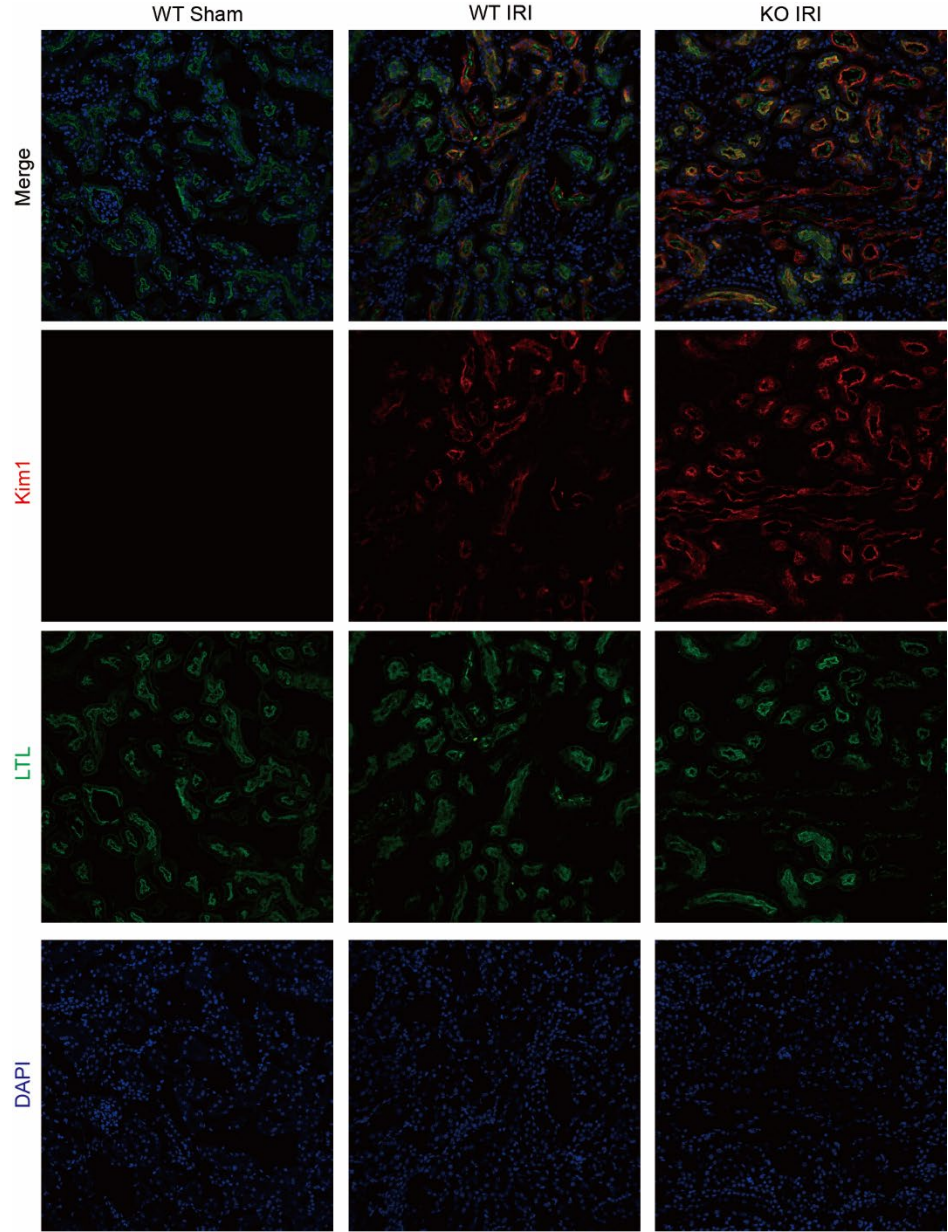

**Fig. 1C**

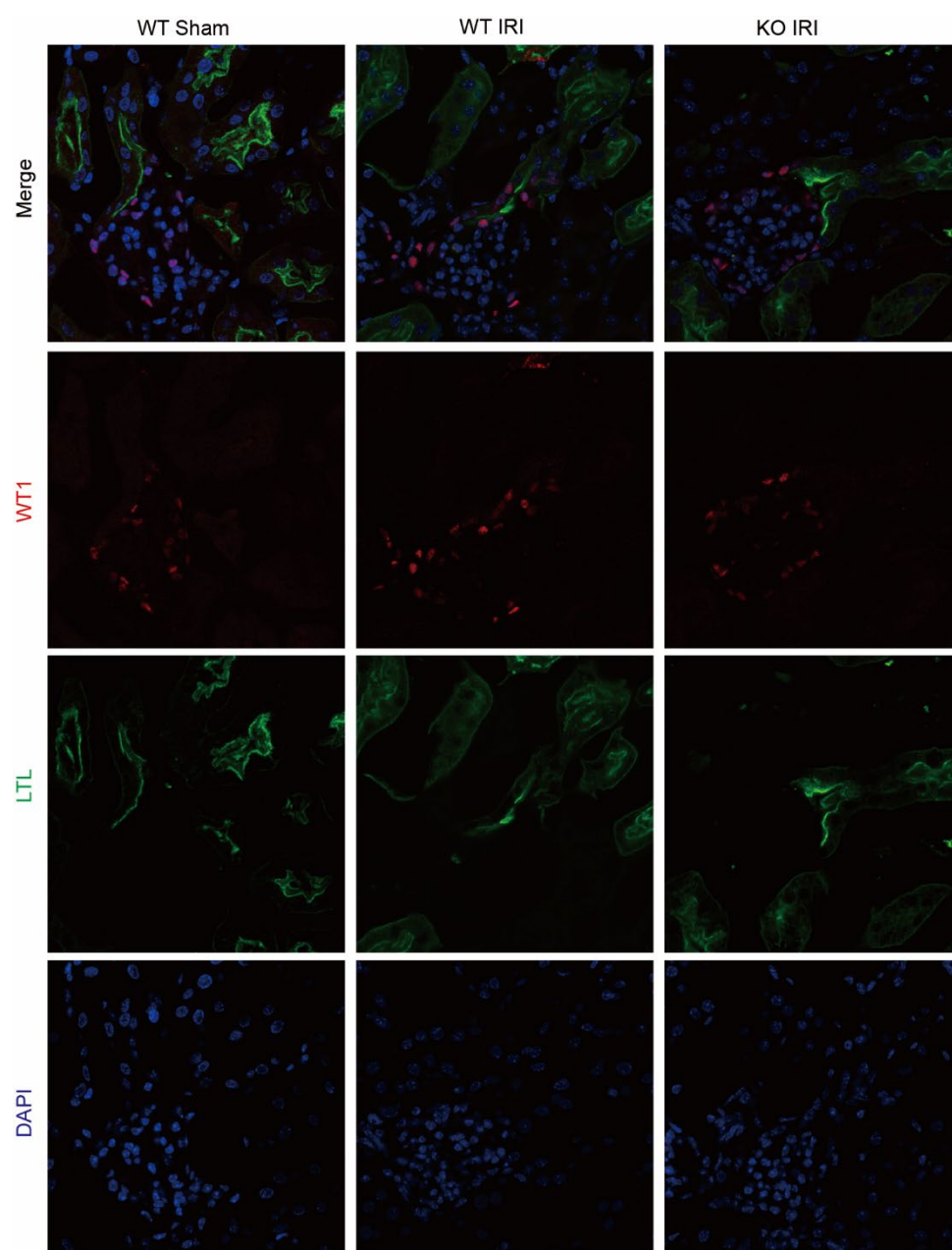

Fig. 7D

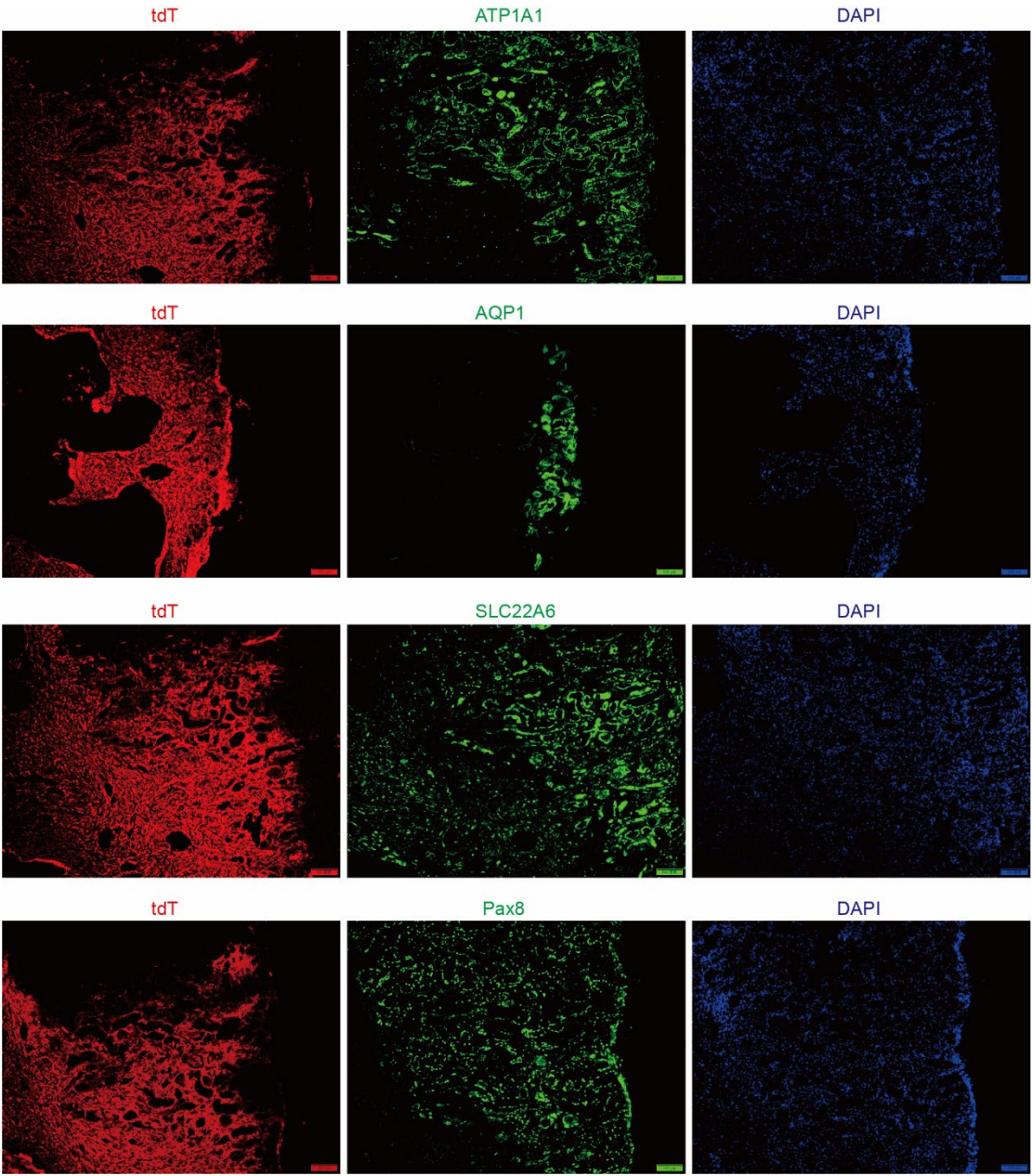

**Fig. S4C**

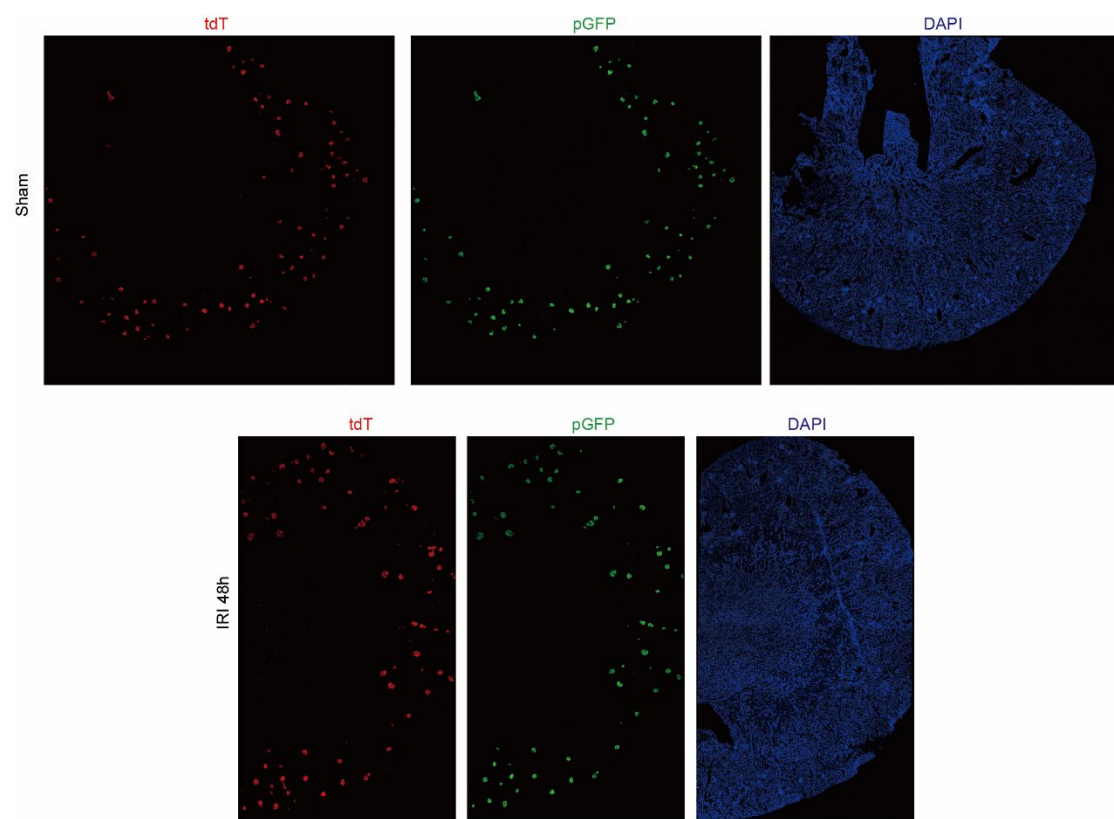

Fig. S4D

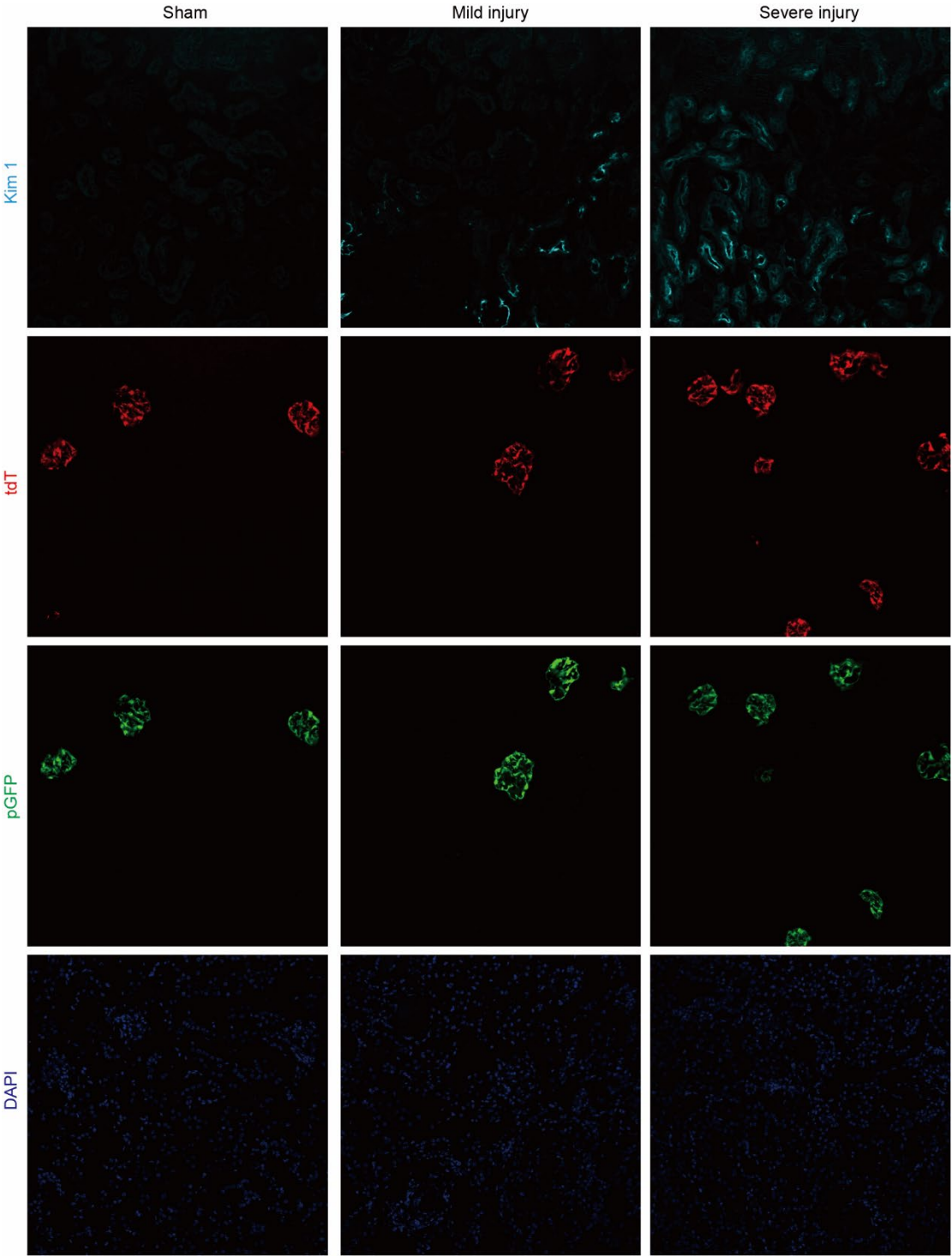

Supplement: Supplementary file 5 — Supplementary raw images. [file thnov13p1311s5.pdf]
